# Technology-enhanced training for prehospital mass-casualty incident preparedness: A scoping review

**DOI:** 10.1371/journal.pone.0348829

**Published:** 2026-11-12

**Authors:** Ameline Saouli, Ali AlRahma, Hadeel Farhan, Abu Omayer, Radwa Nour, Azza Yousif, Ives Hubloue, Nabil Zary

**Affiliations:** 1 College of Medicine, Mohammed Bin Rashid University of Medicine and Health Sciences, Dubai Health, Dubai, United Arab Emirates; 2 Emergency Department, Dubai Health, Dubai, United Arab Emirates; 3 Institute of Learning, Mohammed Bin Rashid University of Medicine and Health Sciences, Dubai Health, Dubai, United Arab Emirates; 4 Emergency Department, UZ Brussel University Hospital, Brussels, Belgium; 5 Research Group on Emergency and Disaster Medicine (ReGEDiM) Vrije Universiteit Brussel, Brussels, Belgium; Khyber Medical University, PAKISTAN

## Abstract

**Background:**

The use of technology-enhanced training for prehospital mass-casualty incident (MCI) preparedness has expanded rapidly, yet how these technologies function throughout the educational process and how competencies are evaluated remain systematically under-examined. This scoping review, conducted as part of the MCIPHER (Mass-Casualty Incident Prehospital Emergency Response) project, mapped the technological modalities, educational functions, and assessment frameworks used in prehospital disaster training.

**Methods:**

Following Arksey and O’Malley’s framework with Levac refinements and PRISMA-ScR reporting, we searched seven databases and supplementary sources for English-language studies and protocols (2015–2025) on technology-enhanced prehospital MCI training for healthcare learners and professionals. Two reviewers independently screened and extracted data. Descriptive and narrative synthesis included modified Kirkpatrick outcome mapping for completed studies, incorporating L2+ (Applied Learning).

**Results:**

Twenty-eight studies met the inclusion criteria. Virtual reality was the most common modality (43%, n = 12), followed by hybrid approaches (29%, n = 8) and screen-based simulations (21%, n = 6). Synthesis of the interventions revealed three consistent operational patterns: half of the studies (50%, n = 14) utilized dual-purpose platforms for both training delivery and automated assessment; embedded data collection was directly linked to achieving applied learning outcomes; and 75% (n = 21) lacked an explicit training design framework. Two additional patterns—a disconnect between immersion level and evaluation depth, and a tension between technological sophistication and scalability—emerged as areas needing future research. Furthermore, no studies originated from low- or lower-middle-income countries. Using the modified Kirkpatrick framework, 56% (n = 14 of 25 completed studies) demonstrated applied learning through embedded performance metrics. Only one study (4%) evaluated workplace behavior change (L3), and none (0%) assessed organizational impact (L4).

**Conclusions:**

The educational value of technology in prehospital MCI training depends more on integrated performance measurement than on visual fidelity or surface immersion. Future investments should prioritize dual-use platforms with embedded assessment, improve pedagogical reporting transparency, and address geographic disparities in training access.

## 1. Introduction

A mass-casualty incident (MCI) refers to an event in which the number and clinical acuity of casualties exceed the available capacity of emergency medical services, resulting in system-wide overload. MCIs may arise from natural or human-made disasters, including terrorist attacks, mass transportation incidents, fires, hazardous material exposure, extreme weather events, and large-scale public health emergencies [1,2].

The present review concerns preparation for the early prehospital phase of MCI response, before hospital-based care, including field triage, ambulance scenarios, roadside emergencies, and disaster zones. It examines training for healthcare learners and professionals across international settings, where the central challenge is coordinating triage, communication, and resource use when local response capacity is exceeded [1,2].

Mass-casualty incidents (MCIs) are among the most complex and mentally demanding situations faced by prehospital care teams. During these events, responders must quickly evaluate and triage many patients, manage limited resources, communicate across various agencies, and make crucial decisions in unstable and risky environments [2]. While MCIs are rare, their impact is significant. Poor preparedness can cause preventable injuries, deaths, and system failures that affect healthcare and communities [3]. Therefore, disaster training for prehospital personnel is essential for strengthening health system resilience and reducing disaster risks worldwide [4].

Although MCI preparedness is crucial, traditional disaster education methods pose notable challenges. Didactic lectures, tabletop drills, and live simulations are still core training tools, but they tend to be resource-heavy, logistically complicated, hard to standardize, and difficult to scale or repeat, especially in remote or resource-limited areas [5]. Additionally, opportunities for repeated realistic MCI practice are scarce because of ethical, safety, and cost issues related to large-scale exercises. This training gap results in many prehospital systems being insufficiently prepared for mass-casualty emergencies [6].

Recent progress in educational technology has opened up new opportunities to bridge this gap. Tools such as virtual reality (VR), augmented and mixed reality (AR/MR), serious games, screen-based simulations, wearable sensors, mobile apps, and digital communication platforms are increasingly used in emergency and disaster medicine training [7,8]. These technologies offer immersive or interactive experiences of disaster scenarios, enabling learners to practice complex situations safely and providing digital records of their actions for feedback, reflection, and assessment. Technology here goes beyond immersive simulation, also including tools for remote learning, performance tracking, communication, and training assessment [9]. While these approaches have great potential to improve accessibility, standardization, and scalability of MCI training, significant evidence gaps still exist.

The review covers literature published during 2015–2025. The 2015 starting point aligns the review with the Sendai Framework for Disaster Risk Reduction 2015–2030 and its emphasis on preparedness [10]. The window was selected to focus on contemporary mobile and immersive technologies and competency-based and simulation-based education, as detailed in the eligibility criteria (Section 2.4.1). Within this period, the practical rationale is to identify how technologies support repeatable training and meaningful competency assessment, rather than simply how they reproduce disaster scenarios.

A recent systematic review examined extended reality technologies, including VR and AR, for MCI training among first responders [9]. However, the existing literature remains incompletely mapped in three important ways. First, prior synthesis has focused mainly on immersive technologies, leaving less clarity on the broader range of digital tools used in MCI education, including serious games, screen-based simulations, mobile applications, wearable sensors, communication devices, and performance dashboards. Second, it remains unclear how these technologies function within the training process: whether they simply deliver scenarios, support learner interaction, provide feedback, capture performance data, or serve as assessment tools. Third, limited attention has been given to how studies report training design frameworks, operational response models, assessment methods, and evaluation levels. As a result, the field lacks a clear overview of how technology-enhanced MCI training is designed, measured, and implemented, and whether these approaches generate evidence of applied competence beyond learner satisfaction or knowledge gain.Global disaster risk reduction frameworks, including the Sendai Framework (Priority 4) [10] and World Health Organization guidelines [11], emphasize simulation exercises and workforce capacity building as essential to health emergency preparedness. While technology-enhanced methods can improve the accessibility, standardization, and scalability of mass-casualty incident (MCI) training, evidence regarding how these technologies function throughout the training cycle and whether they generate measurable skill improvements remains limited and scoping reviews are ideal for addressing these questions by systematically charting diverse literature and identifying evidence gaps without requiring effectiveness synthesis [12]. This scoping review aims to systematically chart how technology is used for training and evaluating prehospital MCI preparedness. Specifically, we seek to (1) identify the technologies used in MCI training and describe their educational roles, such as training delivery, learner interaction, assessment, feedback, and debriefing methods; (2) map reported learner outcomes and measurement strategies, including alignment with frameworks like Kirkpatrick levels; and (3) summarize reported gaps, barriers, and limitations that affect the design, implementation, and scalability of technology-based MCI training. These findings aim to guide future research, support evidence-informed curriculum development, and assist policymakers in enhancing disaster preparedness within prehospital care systems.

## 2. Materials and methods

### 2.1. Study design and methodological framework

This scoping review was part of the MCIPHER (Mass-Casualty Incident Prehospital Emergency Response) project. We followed Arksey and O’Malley’s framework [13], with Levac refinements [14], and PRISMA-ScR reporting guidance [15].

The protocol was prospectively developed, registered on Protocols.io [16], and published on preprints.org [17]. We omitted the optional stakeholder consultation stage because the review aimed to map published literature rather than stakeholder perspectives.

### 2.2. Stage 1: Identifying the research questions

The review was guided by one primary and three secondary research questions.

#### 2.2.1. Primary research question.

What technologies have been used to train and assess healthcare learners and professionals in prehospital mass-casualty incident (MCI) preparedness, and what outcomes have been reported in association with these interventions?

#### 2.2.2. Secondary research questions.

- What learner outcomes are reported (such as knowledge acquisition, triage accuracy and performance, clinical decision-making, time-based performance metrics, stress, or cognitive workload), and how do these outcomes correspond with Kirkpatrick’s levels of evaluation?
- What measurement approaches and instruments are employed (for example, knowledge assessments, performance metrics, structured observational tools, system-generated logs, physiological measures, or usability and acceptability scales)?
- Which training design frameworks are reported, and how explicitly are they described within the included studies?

We organized these questions based on the Population–Concept–Context (PCC) framework. The population included healthcare professionals and trainees; the concept focused on technology-enhanced training or education; and the context was prehospital mass-casualty incidents.

### 2.3. Stage 2: Identification of relevant studies

We developed the search strategy on June 13, 2025, using keywords and Medical Subject Headings (MeSH) informed by preliminary scoping searches and the PubMed MeSH database. Terms covered three components:

- **Population** (for example, “first responders”, “medical intern”, “healthcare provider”, “Paramedics”[MeSH], “Health Personnel”[MeSH], “Nurses”[MeSH], “Physicians”[MeSH])
- **Technology/Concept** (for example, eye-tracking, virtual reality, augmented reality, mixed reality, biometric sensors, wearable devices, digital monitoring, serious games, mobile applications, artificial intelligence, immersive environments, haptic technology, smart wearables, performance dashboards)
- **Disaster/MCI context** (for example “mass casualty incident”, disaster, earthquake, flood, traffic accident, CBRN/CBRNe, terrorist attack, wildfire, multiple trauma, human-made disasters)

We tailored searches for PubMed, Embase, Scopus, CINAHL, PsycINFO, the Cochrane Library, and ClinicalTrials.gov using controlled vocabulary, free-text terms, and Boolean operators. Complete database-specific strategies are provided in S1 Appendix.

The initial search was conducted in June 2025. The strategy was peer-reviewed using PRESS (Peer Review of Electronic Search Strategies) guidelines [18], and searches were revised before final analysis. We also screened Google Scholar and reference lists of included articles and relevant systematic reviews.

### 2.4. Stage 3: Selection of studies

We used Covidence (Veritas Health Innovation, Melbourne, Australia) [19] to import references, remove duplicates, and manage title/abstract screening and full-text review.

#### 2.4.1. Eligibility criteria.

We established eligibility criteria based on the PCC framework. We limited inclusion to studies published from 2015 onward to capture a contemporary evidence base aligned with the Sendai Framework for Disaster Risk Reduction 2015–2030 [10], the wider uptake of current mobile and immersive training technologies [20], and the consolidation of competency-based and simulation-based medical education [21], and applied them consistently during the screening process.

***Inclusion criteria***

- **Population**: Medical first responders, paramedics, emergency medical technicians (EMTs), physicians, nurses, medical interns, residents, and students enrolled in health-related university programs (for example, medicine, nursing, EMS).
- **Concept**: Studies describing, implementing, or evaluating educational or training programs that use any form of technology within the context of MCIs. Eligible technologies included virtual, augmented, or mixed reality (VR/AR/MR), serious games, mobile applications, wearable devices, artificial intelligence-based systems, and high-fidelity simulations incorporating digital components.
- **Context**: Training or simulation activities that replicate prehospital settings, including field triage, ambulance scenarios, roadside emergencies, or disaster zones.
- **Study design**: Original empirical research and study protocols employing quantitative, qualitative, or mixed-methods designs.
- Publication characteristics: Studies available in English were only eligible.
- **Other sources**: Grey literature was eligible if it contained sufficient methodological detail for appraisal and data extraction.

***Exclusion criteria***

- Studies conducted exclusively in in-hospital environments (for example emergency departments, intensive care units, operating rooms) without a prehospital or extramural component.
- Studies focused solely on non-disaster emergency care, routine trauma management, or non-MCI clinical procedures.
- Studies involving only non-healthcare populations (for example, firefighters, police, military, laypersons), unless data pertaining to healthcare professionals were reported separately.
- Secondary research, including systematic reviews, narrative reviews, and scoping reviews, was used for reference list screening.

#### 2.4.2. Study selection process.

Two reviewers independently screened titles and abstracts in Covidence against the predefined eligibility criteria. Records considered potentially eligible by both reviewers proceeded to full-text review.

Two reviewers independently assessed full texts. Disagreements were resolved by discussion, with a third reviewer making the final decision when consensus was not reached.

We recorded full-text exclusion reasons and summarized identification, screening, eligibility assessment, and inclusion in a PRISMA flow diagram.

### 2.5. Stage 4: Data charting

We piloted a standardized extraction form on five studies relevant to technology-enhanced healthcare and emergency training, refining its methodological, educational, and technological fields before final data charting.

The final data extraction form included: study identification, publication year, country, study design, population type, purpose of technology use (training, assessment, or both), technological modality, training design and evaluation frameworks, operational frameworks, data collection instruments, timing of data collection, data type (quantitative, qualitative, or mixed), and reported outcomes mapped to Kirkpatrick levels.

Data were extracted independently in duplicate and reconciled through discussion, with third-reviewer consultation when needed.

#### 2.5.1. Kirkpatrick levels of evaluation.

We categorized outcomes using Kirkpatrick’s four-level Evaluation Model [22]: reaction (Level 1), learning (Level 2), workplace behavior (Level 3), and organizational impact (Level 4).

**Level 1: Reaction**

Outcomes that reflect participants’ immediate reactions to the training, such as satisfaction, perceived engagement, relevance, or confidence reported after the intervention.

**Level 2: Learning**

Outcomes that assess knowledge acquisition, skill development, or attitudinal change are usually measured through structured methods like quizzes, written tests, performance checklists, or validated questionnaires. Self-perceived learning was classified as Level 2 when it was framed as learning gain rather than satisfaction. Eye-tracking results were categorized as Level 2 when interpreted as related to attention or situational awareness.

**Level 2+: Applied Learning**

We introduced a sub-level between Level 2 and Level 3 to distinguish simulation-based performance assessment from real-world behavioral transfer. We classified outcomes as Level 2+ when they met all three of the following criteria: (C1) assessment occurred during or was embedded within the exercise, (C2) performance was measured against an external standard or rubric rather than self-report, and (C3) outcomes reflected the integrated application of knowledge to realistic decisions. L2+ distinguished applied simulation performance from knowledge recall without establishing real-world transfer, which is difficult to assess during actual MCIs for ethical and safety reasons [23,24]. This approach aligns with the companion MCIPHER Tabletop scoping review.

**Level 3: Behavior**

Outcomes reflect the application of learned skills in real-world practice. Because Level 3 is ideally assessed in actual work environments, we used a modified Kirkpatrick approach: we categorized observable simulation-based behaviors (such as triage accuracy, triage time, protocol adherence, communication behaviors) within technology-enhanced settings as practical proxies when real-world exposure is rare [17,18]. We viewed these outcomes as early signs of potential transfer rather than conclusive evidence of actual workplace behavior change.

**Level 4: Results**

Outcomes demonstrating system-level or organizational impact, such as better disaster response metrics, fewer errors, or increased operational efficiency. We excluded studies without measurable organizational outcomes at this level.

When an outcome could potentially fit multiple Kirkpatrick levels, we assigned it to the level that most accurately reflected its main purpose and measurement method.

#### 2.5.2. Framework extraction.

We extracted named or explicitly described frameworks, models, and structured approaches addressing training design, outcome assessment, and operational disaster response. Assessment extraction included both broad evaluation frameworks and standardized instruments when they served as the main outcome measurement method.

### 2.6. Stage 5: Collating, summarizing, and reporting the results

We grouped extracted data by study characteristics, designs and participants; technology purpose and modality; Kirkpatrick outcomes, including L2+; training design, assessment and operational frameworks; and data collection instruments, timing and types.

#### 2.6.1. Data synthesis approach.

We summarized quantitative study characteristics using descriptive statistics, mainly frequencies and proportions. Qualitative and contextual information on training design, implementation, and evaluation was combined through narrative thematic synthesis within and across domains.

Descriptive frequencies used all included records, including completed studies and protocols or registrations, for characteristics available across both groups. Outcome mapping, including Kirkpatrick alignment, was restricted to completed studies reporting measurable outcomes; protocols without outcomes were excluded from this component.

#### 2.6.2. Development of analytical constructs.

Iterative comparison and grouping of studies identified five analytical constructs describing patterns, similarities, and differences across the evidence base.

1. **Technology Function Spectrum** (based on corpus data): We categorized technologies along a range from delivery-only (content transmission without assessment) to dual-use (simultaneous training and assessment), and assessment-focused (mainly designed for performance measurement).
2. **Data Capture Architecture** (corpus-derived): We explained how outcomes were recorded: embedded within the technology (system-generated metrics), outside the technology (observer-rated or manual collection), or multimodal (combining multiple data sources).
3. **Pedagogical Transparency Gap** (corpus-derived): We assessed how well studies clearly aligned their training design, assessment methods, and operational models taught. Studies with high transparency detailed all three areas; those with low transparency left out or poorly linked one or more.
4. **Immersion-Evaluation Paradox** (hypothesis-generating): We noticed a conflict between the level of immersion in the training setting and the thoroughness of the assessment. High-immersion technologies often lacked strong evaluation methods, while standardized assessment tools sometimes operated in less immersive environments.
5. **Scalability-Rigor Tension** (hypothesis-generating): We identified recurring trade-offs between technology sophistication and practical implementation capacity. Complex, high-fidelity systems enable rigorous measurement but require significant resources; simpler technologies are easier to implement but provide less granular outcome data.

The first three constructs represent systematically observed corpus features. The final two are hypothesis-generating patterns requiring testing in future implementation studies.

Operational definitions and rules for study-level construct assignment are provided in S2 Appendix.

### 2.7. Ethics

Ethical approval was not required because the review used published, publicly available data without direct participant contact. We followed ethical research standards for transparent reporting, accurate citation, and avoidance of plagiarism or duplicate publication, and managed potential conflicts of interest according to institutional policies.

## 3. Results

### 3.1. Literature search and screening

We identified 2,652 records from database searches (Embase, PubMed, Scopus, CINAHL, Cochrane Library, PsycINFO, and ClinicalTrials.gov) and 9 additional records from other sources. After removing 556 duplicates, we screened 2,105 records by title and abstract. We requested 173 full reports; 25 could not be retrieved. We assessed 148 full-text reports for eligibility and excluded 120 due to incorrect setting, concept, context, population, study design, or non-English language. We included 28 studies in the final synthesis [25–52]. Fig 1 shows the PRISMA flow diagram.

**Fig 1:**
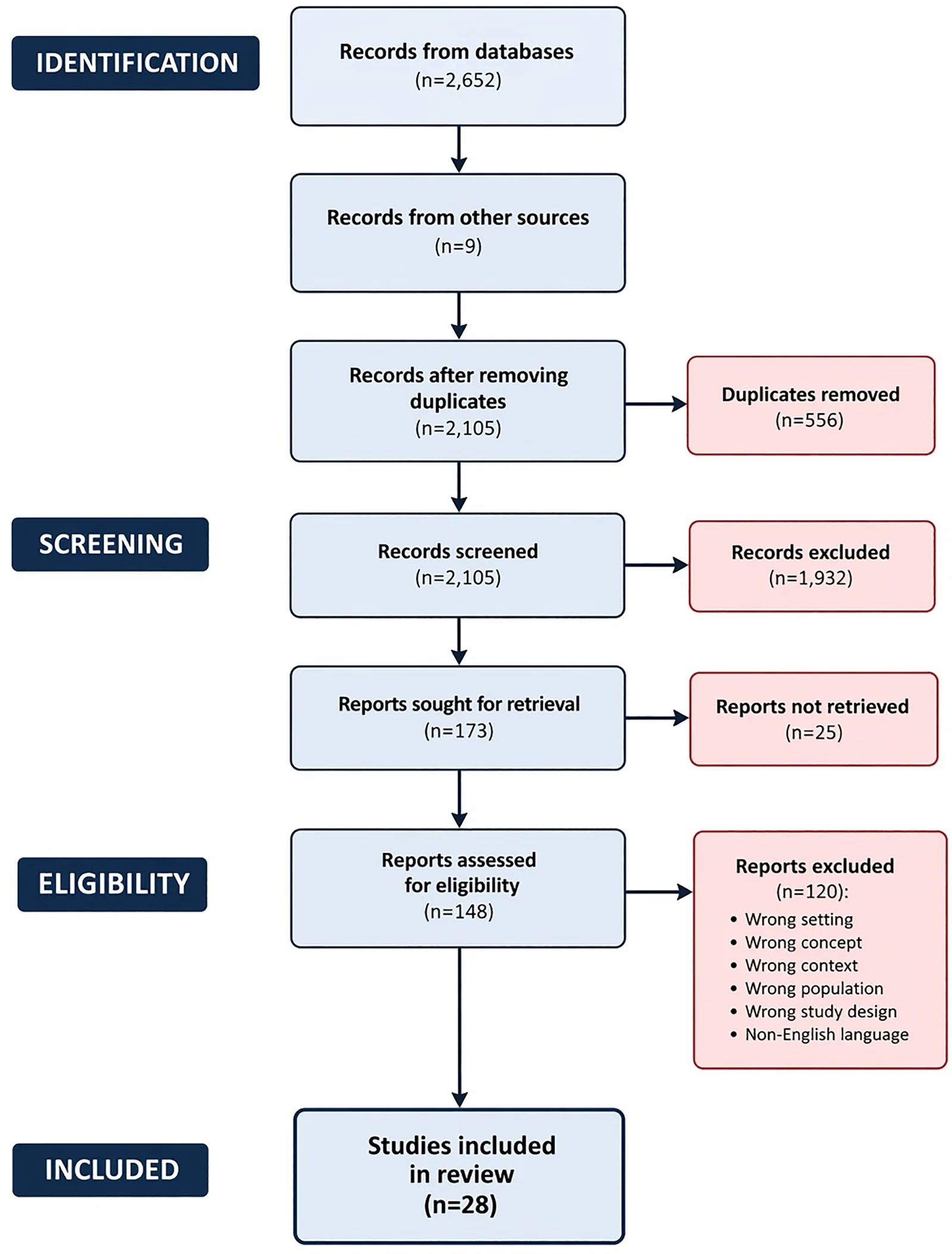
PRISMA 2020 flow diagram for the scoping review of technology-enhanced training for prehospital mass-casualty incident preparedness.

*Records were identified from seven electronic databases (Embase, PubMed, Scopus, CINAHL, Cochrane Library, PsycINFO, and ClinicalTrials.gov) and supplementary sources (Google Scholar and reference list screening). A total of 28 studies met all inclusion criteria and were included in the final synthesis.*

### 3.2. Study characteristics

The 28 included studies were mainly published within the past three years. The United States contributed the most publications (7 studies), followed by Sweden (3 studies), while Canada, China, Germany, and Iran each contributed two studies. The remaining studies originated from Belgium, the Czech Republic, England, Hong Kong, Jordan, Saudi Arabia, South Korea, Taiwan, Thailand, and Turkey. Based on World Bank income classifications, 20 studies (71%) were conducted in high-income countries and 8 (29%) in upper-middle-income countries (China, Iran, Thailand, Turkey, Jordan). No studies were conducted in low- or lower-middle-income countries (Table 1).

**Table 1:** Study Characteristics.

Study ID	Study	Year	Country	Study Design	Population	Sample Size	Technology Type
1	Goldberg 2021	2021	USA	RCT	Pediatric EM physicians, fellows, residents	50	Communication devices (goTenna)
2	McCoy 2019	2019	USA	Mixed-methods	Physicians, nurses, EMTs, paramedics	32	Smart glasses (Google Glass), telesimulation
3	Cicero 2017	2017	USA	RCT	Paramedics, paramedic students, EMTs	47	Video game/serious game
4	ChiCTR2300072282 2023	2023	China	RCT protocol	Nurses	160	Virtual reality
5	Chumvanichaya 2025	2025	Thailand	RCT	Paramedic students	83	Virtual reality
6	Hosseini 2023 (2)	2023	Iran	Quasi-experimental	Nursing students	60	Game-based training
7	Way 2024	2024	USA	Mixed-methods	Paramedics, EMTs, medical students, EM physicians	375	VR (Meta Quest 2)
8	Heldring 2025	2025	Sweden	Mixed-methods	Ambulance nurses, RNs, nursing students, EMTs	95	VR (HTC VIVE, GoSaveThem)
9	Shujuan 2022	2022	China	RCT	Nursing students	101	Virtual reality
10	Jain 2016	2016	Canada	Prospective cohort	Paramedic students	26	VR simulation (XVR)
11	Cicero 2019	2019	USA	RCT	Paramedics, EMTs	26	Immersive + screen-based simulation
12	Baetzner 2025	2025	Germany	Quasi-experimental	Paramedics, EM physicians, medical students, nurses	76	VR (XVR + Varjo Aero) + eye-tracking
13	Hermann 2021	2021	Germany	Pre-post evaluation	Medical students	102	Computer-based simulation
14	Kyoung 2023	2023	South Korea	Usability study	Nursing students	30	Virtual reality
15	Hosseini 2023 (1)	2023	Iran	Quasi-experimental	EM students	120	Screen-based simulation (SIG)
16	NCT06253156 2024	2024	Turkey	RCT protocol	Nursing students	67	Virtual reality
17	NCT06034184 2024	2024	Sweden	RCT protocol	Nursing students	60	Virtual reality
18	Wetherell 2024	2024	England	Mixed-methods	EM/ICU physicians, paramedics	15	Smartwatches (Garmin)
19	Hu 2024	2024	Hong Kong	Quasi-experimental	Nurses	106	Computer game simulation
20	Alhawatmeh 2025	2025	Jordan	RCT	Paramedic students	102	Immersive VR
21	Bauchwitz 2024	2024	USA	Quasi-experimental	Medical students, paramedics, nurses, EM residents/attendings	21	Smartphone simulation (EFECTIVE)
22	Chevalier 2023	2023	Belgium	Cross-sectional	Ambulance attendants, nursing students, medical students	83	Virtual reality
23	Heldring 2024	2024	Sweden	Mixed-methods	Ambulance clinicians	11	VR (HTC VIVE, GoSaveThem)
24	Lochmannová 2025	2025	Czech Republic	Mixed-methods	Paramedic students	37	VR + Garmin smartwatches
25	Sibley 2018	2018	Canada	Intervention (post-test)	EMTs, ED nurses, ED physicians	96	UAV/drone
26	Chang 2022	2022	Taiwan	Quasi-experimental	ED nurses	67	360° VR (HTC VIRTI)
27	Foronda 2016	2016	USA	Pre-post evaluation	BSN students	6	Web-based 3D simulation (V-CAEST)
28	Bajow 2016	2016	Saudi Arabia	Pre-post evaluation	Medical students	29	XVR, ISEE, video lectures/e-learning

Characteristics of the 28 included studies on technology-enhanced training for prehospital mass-casualty incident preparedness. Studies are presented by study ID. Sample sizes reflect reported participant numbers; for protocols, planned enrollment is indicated. Country refers to the location where the study was conducted. Technology type describes the primary technology platform used.

Study designs varied considerably. Six studies (21.4%) each employed randomized controlled trials (RCT), mixed-methods, and quasi-experimental designs. Three studies (10.7%) were RCT protocols, and three (10.7%) used pre-post designs. The remaining studies included one cross-sectional study, one intervention study (post-test only), one prospective cohort study, and one usability study, each representing 3.6% (Table 2).

**Table 2:** Study Design Frequencies.

Study Design	Number of Studies	Percentage
Mixed-methods	6	21.4%
Quasi-experimental design	6	21.4%
Randomized Controlled Trial (RCT)	6	21.4%
RCT protocol	3	10.7%
Pre-post evaluation	3	10.7%
Cross-sectional study	1	3.6%
Intervention study (post-test only)	1	3.6%
Prospective cohort study	1	3.6%
Usability study	1	3.6%
**Total**	**28**	**100%**

Distribution of study designs across the 28 included studies. Percentages are calculated from the total number of included studies (N = 28). Three studies were registered protocols reporting planned methodology without outcome data.

Participants came from various healthcare roles. Nursing students made up the largest group (32.1% of studies), followed by paramedics, physicians, and nurses (25% each). The physician and trainee groups included pediatric emergency medicine specialists, emergency medicine residents, anesthesiologists, and critical care providers. The diverse specialties highlight the wide relevance of MCI training across healthcare systems.

### 3.3. Data types and collection methods

We documented a variety of quantitative, qualitative, and mixed-method data collection approaches across the 28 studies.

**Quantitative measures** were predominant. Pre- and post-test assessments appeared in 11 studies. Multiple-choice knowledge questions were included in 6 studies. Triage accuracy and performance metrics, such as speed, accuracy, and error rate, were documented in 8 studies. Structured questionnaires, including the State-Trait Anxiety Inventory (2 studies), NASA Task Load Index (2 studies), and technology acceptance scales (3 studies), assessed psychological and usability outcomes.

**Performance-based data sources** appeared in 14 studies. VR systems logged learner actions and decisions (5 studies). Game databases captured learning events and performance data (2 studies). Eye-tracking outputs measured visual attention patterns (1 study). Smartwatch biometrics and salivary cortisol sampling captured physiological stress (2 studies). Audio and video recordings enabled later coding and analysis (3 studies).

**Qualitative methods** were used in 8 studies. Interviews and chart-stimulated recall examined cognitive processes and decision-making (2 studies). Open-ended survey questions measured participant perceptions (3 studies). Debriefing notes recorded facilitator observations (1 study). Observational tools systematically tracked participant behaviors (2 studies).

**Mixed-methods designs** were used in 6 studies, typically combining surveys with performance metrics or observational data. This combination allowed for triangulation between subjective experiences and objective performance measures.

Comprehensive instrument-by-instrument documentation is available in S4 Appendix, which outlines data types, data collection timing, and study-specific modifications.

### 3.4. Technologies and their educational functions

Twelve studies (42.9%) primarily used virtual reality (VR) as the main technology modality. Eight studies (28.6%) combined multiple formats, such as video, simulation, and in-person approaches, into hybrid methods. Six studies (21.4%) utilized screen-based simulations. Two studies (7.1%) involved hands-on or fully in-person, technology-supported training. Detailed specifications and applications for each study are listed in Table 3, with additional classifications provided in S3 Appendix.

**Table 3:** Technology Applications.

Study ID	Study	Technology Modality	Purpose	Function Spectrum
1	Goldberg 2021	In-person (communication devices)	Training	Hybrid
2	McCoy 2019	Hybrid (smart glasses + telesimulation)	Both	Hybrid
3	Cicero 2017	Screen-based (serious game)	Training	Dual-use
4	ChiCTR2300072282 2023	VR	Training	Delivery-only
5	Chumvanichaya 2025	VR	Training	Dual-use
6	Hosseini 2023 (2)	Screen-based (game-based)	Training	Delivery-only
7	Way 2024	VR (Meta Quest 2)	Both	Dual-use
8	Heldring 2025	VR (HTC VIVE)	Both	Dual-use
9	Shujuan 2022	VR	Both	Dual-use
10	Jain 2016	VR (XVR)	Training	Dual-use
11	Cicero 2019	Hybrid (immersive + screen)	Training	Dual-use
12	Baetzner 2025	VR (XVR + eye-tracking)	Both	Dual-use
13	Hermann 2021	Screen-based + in-person	Both	Delivery-only
14	Kyoung 2023	VR	Training	Delivery-only
15	Hosseini 2023 (1)	Screen-based (SIG simulation)	Both	Delivery-only
16	NCT06253156 2024	VR + in-person	Training	Delivery-only
17	NCT06034184 2024	VR + in-person	Training	Delivery-only
18	Wetherell 2024	In-person (smartwatches)	Assessment	Assessment-dedicated
19	Hu 2024	Screen-based (game simulation)	Both	Dual-use
20	Alhawatmeh 2025	VR + in-person	Training	Delivery-only
21	Bauchwitz 2024	Screen-based (smartphone)	Training	Dual-use
22	Chevalier 2023	VR	Training	Dual-use
23	Heldring 2024	VR (HTC VIVE)	Both	Dual-use
24	Lochmannová 2025	VR + smartwatches	Both	Hybrid
25	Sibley 2018	Screen-based (UAV/drone)	Assessment	Assessment-dedicated
26	Chang 2022	VR (360°)	Training	Dual-use
27	Foronda 2016	Screen-based (web-based 3D)	Training	Dual-use
28	Bajow 2016	Hybrid (XVR + ISEE + e-learning)	Training	Hybrid

Technology applications and functional classification of the 28 included studies. Technology modality describes the primary delivery platform. Purpose of technology indicates whether the system was used for training delivery, assessment, or both. Technology Function Spectrum classifies each study according to the emergent analytical construct: delivery-only (content transmission without embedded assessment), dual-use (simultaneous training and data capture), assessment-dedicated (primarily designed for performance measurement), or hybrid (multi-modal systems combining elements across categories).

### 3.5. Technology function spectrum

Beyond modality, we categorized how technology operated throughout the training cycle. We identified three separate operational models along with a hybrid category.

**Delivery-only systems** [29,30,33,34,36,45,46,49] served as content delivery channels without integrated performance capture. These systems showed scenarios, provided instructional content, or delivered simulations, but depended on external questionnaires or separate assessments to measure outcomes. Examples include VR glasses used only for scenario presentation and computer-based e-learning platforms.

**Dual-use systems** [25–27,31,32,35,38,41–44,50–52] operated simultaneously as training delivery and data collection tools. The technology platform itself recorded learner actions, decisions, timing, and performance metrics. Examples include VR systems that logged triage decisions and timing, game platforms that automatically captured learning events, and screen-based simulations that tracked user interactions through system logs. This integration allowed for real-time feedback, performance analytics, and proof of learning within a single platform.

**Assessment-dedicated systems** [28,47] were used mainly for measuring performance rather than delivering instruction. The wearable technology (smartwatches) tracked physiological stress responses during separate training sessions, and the aerial drone system allowed for scene size-up and hazard identification without providing explicit instructional content.

**Hybrid or multi-modal systems** [37,39,40,48] integrated elements across these categories, combining delivery, data capture, and assessment through multiple coordinated technologies. These studies represent the most complex technology configurations in the corpus.

### 3.6. Data capture architecture: Embedded, external, and multimodal approaches

How technology directly impacted the depth of performance evidence and its effect on outcomes.

**Embedded capture systems** (12 studies) utilized automatic logging within the training platform. VR system logs, game databases, and screen-based simulation platforms recorded learner actions, triage decisions, response times, and error patterns without needing separate assessment tools. This method produced continuous, detailed performance data throughout training.

**External capture systems** (11 studies) used separate assessment tools administered independently of the training technology. Pre- and post-tests, multiple-choice knowledge questions, questionnaires (such as the State-Trait Anxiety Inventory and NASA Task Load Index), and technology acceptance scales were conducted as separate evaluation events. These tools measured knowledge, confidence, workload, or satisfaction but remained disconnected from the training platform.

**Multimodal capture systems** [39,41,43,44,51] combined embedded platform data with physiological measures or external assessments. Smartwatch biometrics paired with VR performance logs, eye-tracking combined with triage accuracy metrics, or haptic feedback synchronized with performance data all produced richer datasets linking behavioral, physiological, and cognitive outcomes.

Out of 17 studies using embedded or multimodal capture, 14 achieved L2+ classification (see below). Among 11 studies relying only on external capture, none reached L2+.

### 3.7. Training outcomes: L2+ Reclassification and Kirkpatrick levels

We applied Kirkpatrick’s four-level evaluation framework to all 28 studies. Outcomes focused on lower levels, prompting a new analytic distinction. Fig 2 shows publication trends over time and the evaluation depth achieved across included studies.

**Fig 2:**
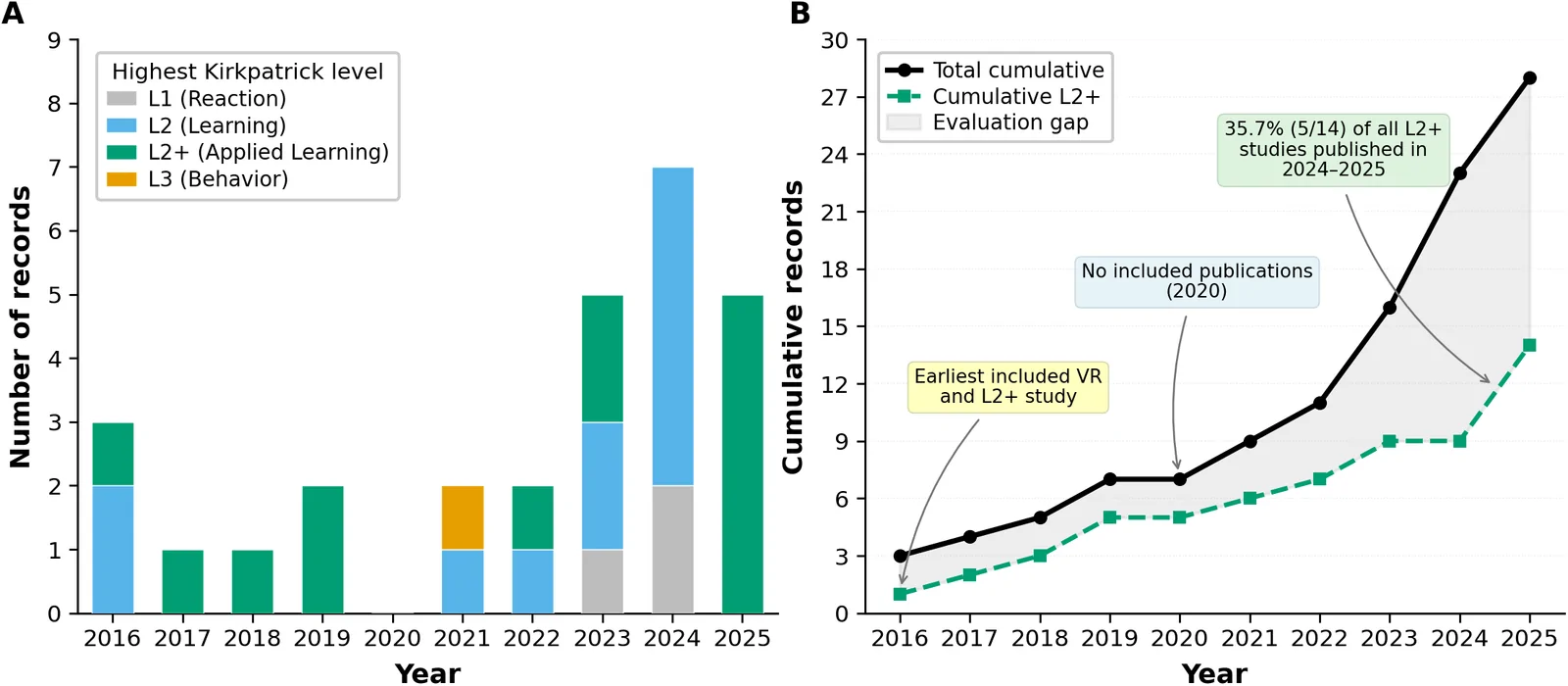
Publication trends and evaluation depth of technology-enhanced MCI training studies (2016-2025). Panel A: Annual publication counts stratified by highest Kirkpatrick evaluation level achieved per study. Colors indicate L1 only (gray), L2 (light blue), L2+ Applied Learning (green), and L3 Behavior (amber). Panel B: Cumulative number of included studies (solid line) and cumulative number of studies achieving L2+ classification (dashed line), with the shaded area representing the evaluation gap. Annotations mark the COVID-19 publication gap (2020) and key milestones. Note the sharp increase in publication volume from 2023, with 2025 studies demonstrating exclusively L2+ evaluation depth.

**Level 1 (Reaction)**: Seventeen studies (61%) reported participant satisfaction, engagement, or usability outcomes through satisfaction surveys and technology acceptance scales.
**Level 2 (Learning)**: Twenty studies (71.4%) evaluated knowledge gain or procedural skills using pre- and post-tests, multiple-choice questions, or performance measures like triage accuracy.
**Level 3 (Behavior)**: Only 1 study provided evidence of behavior change in clinical settings.
**Level 4 (Results)**: No study reported organizational or patient-level outcomes.

Within Level 2, we established a new classification: **L2+ (Applied Learning)**. This subcategory includes studies that demonstrated competency through authentic, performance-based assessments under conditions similar to real-world MCI response. L2+ required integrated data collection within realistic simulation scenarios, providing objective evidence of skill application rather than just knowledge recall.

Among the 25 completed studies, 14 (56%) met all three L2+ criteria [25–27,30–33,39–41,44,47,48,51]. Two protocols [36,49] describe methods consistent with L2+ criteria pending outcome reporting (Table 4).

**Table 4:** Kirkpatrick Evaluation Levels with L2+ Reclassification.

Study ID	Study	L1 (Reaction)	L2 (Learning)	L2+ (Applied)	L3 (Behavior)	L4 (Results)	Key Outcomes
1	Goldberg 2021	✓	—	✓	✓	—	Communication accuracy, triage accuracy, workload
2	McCoy 2019	✓	✓	✓	—	—	Triage accuracy, satisfaction, self-reported improvement
3	Cicero 2017	—	—	✓	—	—	Triage accuracy
4	ChiCTR2300072282 2023	✓	✓	—	—	—	Attitudes, preparedness
5	Chumvanichaya 2025	✓	✓	✓	—	—	Knowledge, triage accuracy and time, motivation
6	Hosseini 2023 (2)	✓	✓	—	—	—	Satisfaction, attitude
7	Way 2024	✓	✓	—	—	—	Perceived realism, perceived learning
8	Heldring 2025	✓	✓	✓	—	—	Attitude change, triage accuracy, triage time
9	Shujuan 2022	—	✓	✓	—	—	Knowledge, attitude, performance skills
10	Jain 2016	—	—	✓	—	—	Time to triage, triage prioritization accuracy
11	Cicero 2019	—	—	✓	—	—	Triage accuracy
12	Baetzner 2025	—	✓	✓	—	—	Visual attention, triage accuracy, triage speed
13	Hermann 2021	✓	✓	—	—	—	Knowledge, attitude, satisfaction
14	Kyoung 2023	✓	—	—	—	—	Usability
15	Hosseini 2023 (1)	—	✓	✓	—	—	Knowledge, triage performance
16	NCT06253156 2024	—	✓	—	—	—	Attitude change, disaster preparedness
17	NCT06034184 2024	—	✓	—	—	—	Knowledge, triage performance (protocol)
18	Wetherell 2024	✓	—	—	—	—	Anxiety, workload, stress
19	Hu 2024	✓	✓	—	—	—	Knowledge, usability, motivation
20	Alhawatmeh 2025	—	✓	✓	—	—	Knowledge, triage performance
21	Bauchwitz 2024	✓	—	—	—	—	Usability, fidelity, time pressure
22	Chevalier 2023	✓	✓	✓	—	—	Knowledge, triage accuracy, triage time, stress
23	Heldring 2024	✓	✓	—	—	—	Perceived usefulness, perceived learning
24	Lochmannová 2025	✓	—	✓	—	—	METHANE reporting, triage performance, workload
25	Sibley 2018	—	✓	✓	—	—	Knowledge, triage
26	Chang 2022	—	✓	—	—	—	Self-assessed disaster preparedness, self-efficacy
27	Foronda 2016	✓	✓	—	—	—	Satisfaction, knowledge
28	Bajow 2016	✓	✓	—	—	—	Satisfaction, knowledge, self-reported behavior
	**TOTAL**	**17 (61%)**	**20 (71.4%)**	14 (56%*)	**1 (4%)**	**0 (0%)**	*NB: L2+ calculated from 25 completed studies

Kirkpatrick evaluation levels with L2+ (Applied Learning) reclassification for the 28 included studies. Checkmarks (✓) indicate that the study reported outcomes at the corresponding level; dashes (—) indicate no outcomes at that level. L2+ classification required meeting all three criteria: (C1) assessment embedded within the exercise, (C2) performance measured against an external standard, and (C3) integrated application of knowledge to realistic decisions. L2+ percentages are calculated from the 25 completed studies (excluding 3 protocols). Goldberg 2021 is the only study achieving Level 3 (behavior transfer in real-world settings). No study achieved Level 4 (organizational impact).

L2+ classification was based on three criteria: (C1) assessment took place during or was integrated into the exercise, (C2) performance was evaluated against an external standard or rubric rather than self-report, and (C3) outcomes demonstrated the combined application of knowledge to realistic decisions. Studies meeting all three criteria were categorized as L2+.

### 3.8. Pedagogical transparency gap

We identified significant gaps in how studies reported their educational frameworks.

**Operational frameworks** were reported by 22 studies (79%). START triage was reported in 11 studies (39%), either alone or in combination with other systems. JumpSTART appeared in 5 studies (18%). SALT, SIEVE/SORT, and STM each appeared in multiple studies. Broader operational structures, including the Incident Command System, the Sphere Project, and WHO guidance, appeared in 7 studies (25%). This consistent reporting enabled evaluation of whether the training aligned with recognized disaster response protocols.

**Assessment frameworks** were explicitly reported in only 9 studies (32%). These included named instruments such as the NASA Task Load Index, the State-Trait Anxiety Inventory, and the ARCS Motivation Survey, as well as cognitive task analysis frameworks such as the Methodology for Annotated Skill Trees and specialized assessment tools such as the START-Assessment Global Scale and the Sense of Presence questionnaire. Most studies described data collection methods but did not reference a specific assessment framework.

**Training design frameworks** were identified in only 7 studies (25%). Explicit references included Backward Curriculum Design (1 study), Keller’s ARCS Model combined with the System Development Life Cycle (1 study), ADDIE instructional design (1 study), game-based learning principles (1 study), and the Clinical Reasoning Cycle with reflective practice (1 study). However, 21 studies (75%) did not mention any training design model or learning theory, instead focusing on activities or content without an explicit design structure.

**Full framework integration** across all three categories: training design, assessment, and operational, appeared in only 3 studies (11%) [26,34,35].

### 3.9. Cross-cutting patterns: Immersion-evaluation paradox and scalability-Rigor tension

We identified two hypothesis-generating patterns across technology choices and outcome structures.

#### 3.9.1. The immersion-evaluation paradox.

Thirteen studies (46.4%) used immersive VR technology, but immersion level did not predict the depth of evaluation. Studies combining immersive VR with external-only data collection [34,38] only achieved Level 1–2 outcomes despite high fidelity. In contrast, non-immersive screen-based systems with integrated data collection [25,32,44] reached L2+. Data architecture, rather than immersion level, determined the outcome depth.

#### 3.9.2. Scalability-Rigor tension.

Higher-fidelity multi-modal technologies (VR + eye-tracking + physiological monitoring) produce richer performance data but are mainly used in high-resource settings (United States, Sweden, Germany). Lower-cost, more scalable technologies (screen-based simulations, smartphone apps, game-based learning) appear in upper-middle-income settings but often depend on external-only data collection, resulting in Level 1–2 outcomes. This pattern indicates a tradeoff between technological sophistication and implementation reach (Fig 3).

**Fig 3:**
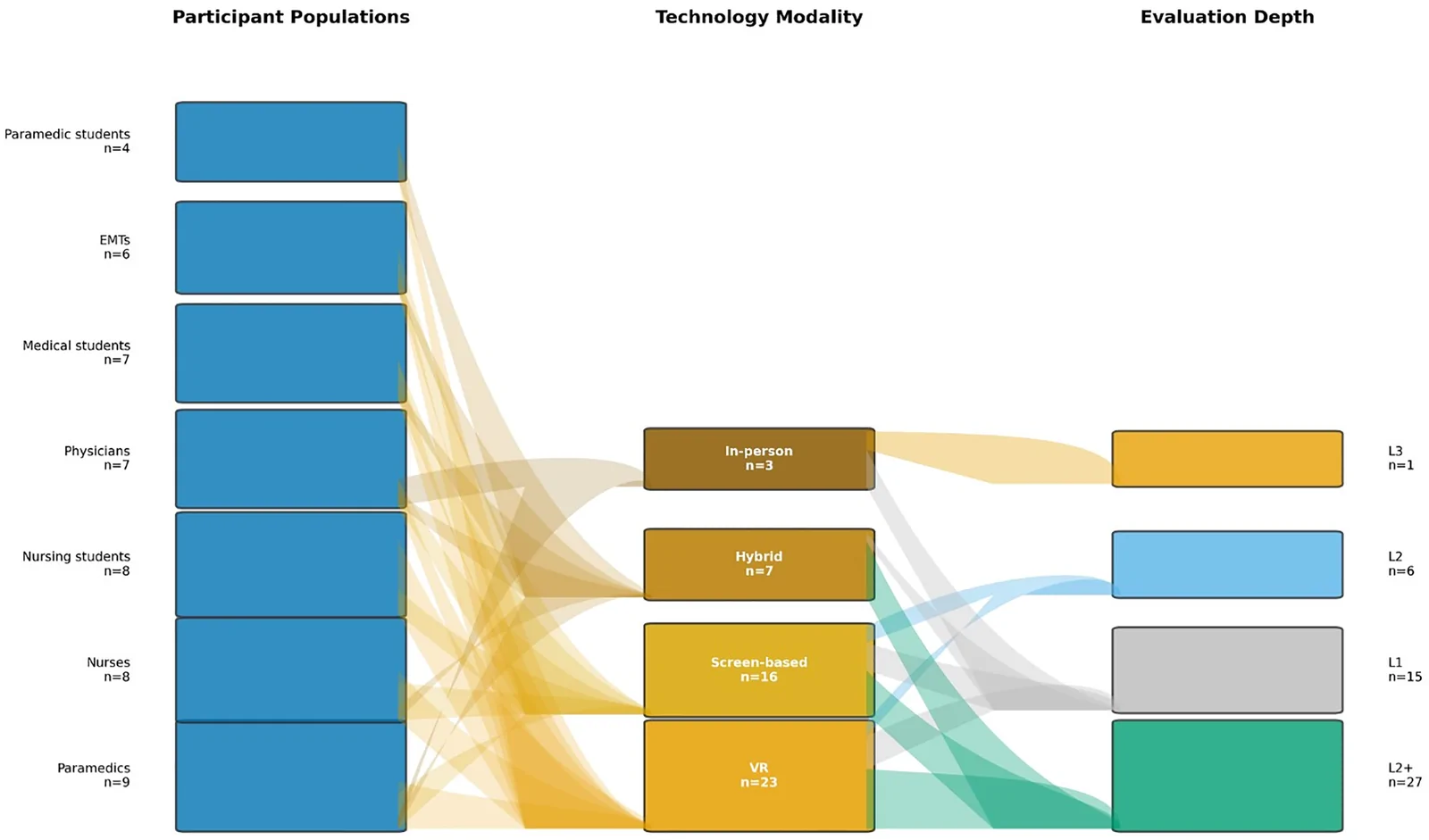
Alluvial diagram illustrating the flow from participant populations (left) through technology modalities (center) to evaluation depth achieved (right) across the 28 included studies. Bandwidth is proportional to the number of study-population pairs (n = 49; studies with multiple participant types contribute multiple flows). Left column categories reflect the seven major population groups identified across the corpus. The center column shows the four technology modality categories. The right column displays the highest Kirkpatrick level achieved per study: L1 Reaction only (gray), L2 Learning (light blue), L2+ Applied Learning (green), and L3 Behavior (amber; Goldberg 2021 only). The diagram visually demonstrates the Immersion-Evaluation Paradox: VR dominates the technology column but splits between L2+ and lower-level outcomes, while screen-based systems show strong flow toward L2+, reflecting the primacy of data-capture architecture over immersion level.

## 4. Discussion

This scoping review mapped the literature on technology-enhanced prehospital mass-casualty incident (MCI) training and identified four distinct empirical patterns across the evidence base. First, technologies that embed real-time performance capture were consistently associated with higher-level applied learning outcomes than platforms used primarily for scenario delivery. Second, evaluation practices remained heavily concentrated at the lower Kirkpatrick levels, with minimal investigation of behavioral transfer and a complete absence of organizational outcome assessment. Third, operational incident-management models were reported with significantly greater consistency than pedagogical designs or assessment frameworks. Finally, the evidence base showed a pronounced geographic concentration in higher-resource settings. Rather than attempting to determine which specific technological modality is inherently superior, this discussion synthesizes these structural patterns through a functional lens—examining how data-capture architectures, instructional transparency, and implementation contexts collectively shape the true educational utility of disaster training platforms.

### 4.1. The function-measurement nexus: Why embedded assessment matters more than surface immersion

A central pattern in the corpus was the contrast between technology used primarily to deliver scenarios and technology used both to deliver scenarios and capture performance. In this review, dual-use systems were more often linked to L2+ outcomes than delivery-only systems, suggesting that the educational role assigned to a platform may matter more than the platform category itself [9,12,53]. Technologies that document decisions, timing, communication, or error patterns provide a stronger basis for judging applied learning than platforms primarily optimized for scenario presentation [25,27,48].

The same pattern emerged when studies were examined in terms of their data-capture architecture. Embedded and multimodal systems more often generated deeper evidence of evaluation because they drew on learners’ actions during the exercise rather than on recall-based or self-report measures administered afterward [35,40,44,50]. In this corpus, measurement capacity appeared to be more closely aligned with evaluation depth than with visual sophistication alone. Some highly immersive systems were evaluated primarily through questionnaires, whereas some less immersive systems yielded richer evidence of applied learning because they captured performance directly [54,55]. The practical implication is therefore not that immersion lacks value, but that immersion alone did not guarantee stronger evidence. For educators and program leaders, systems that combine scenario delivery with embedded assessment may offer a more defensible educational investment than systems that require all evaluation to occur outside the training environment [10–12].

### 4.2. Pedagogical transparency and evaluation depth

The review showed a clear asymmetry in reporting: operational response frameworks were described more often than educational design frameworks. Many studies named the triage or incident-management model being taught, which made the intended operational content visible, but did not make the pedagogical strategy equally transparent [37,44,46,56]. When studies do not specify how objectives were sequenced, how learners were supported, how feedback and debriefing were structured, or how assessment was aligned with the instructional design, it becomes difficult to judge whether observed effects are attributable to the technology, the pedagogy, or both [54,55,57].

The small number of studies that did report an explicit design framework are instructive because they linked learning objectives to scenario structure, feedback processes, and assessment logic, making the intervention easier to interpret and reproduce [34,57]. This broader concern is consistent with simulation and instructional design literature showing that clearer alignment among objectives, activities, assessment, and feedback improves interpretability and transfer [54,55,57]. In the present corpus, incomplete pedagogical reporting also helps explain the Kirkpatrick distribution: most studies assessed learner reaction or learning, very few approached behavioral transfer, and none addressed organizational results.

Within that landscape, the L2+ category remains useful. In this review, L2+ marks studies that moved beyond tests or self-report by assessing decisions against an external standard during scenario performance. Because real-world disaster transfer is difficult to observe directly, L2+ functions as a transparent intermediate standard for applied performance rather than a substitute for Level 3 or Level 4 evidence [12,57]. Reaction outcomes still matter because usability and engagement can affect adoption, but they are not sufficient proxies for preparedness or performance [58]. The practical value of L2+ is that it makes applied performance more visible than reaction-only or knowledge-only approaches do [58].

### 4.3. The scalability-Rigor tension and disaster equity

A major limitation of the evidence base is its concentration in higher-resource settings. Most studies came from high-income countries, none came from low- or lower-middle-income settings, and the most technologically sophisticated approaches were also the least tested for broader system-level transferability [10,11]. This matters because disaster preparedness is also an equity issue: a literature base drawn mainly from higher-resource environments risks widening, rather than narrowing, preparedness disparities [59].

Analogous educational-technology literature identifies familiar adoption barriers, including infrastructure constraints, digital literacy demands, connectivity limitations, and the need for sustained technical support [59,60]. The tension between scalability and rigor is therefore not only methodological but also practical and policy-relevant. Higher-fidelity multimodal systems may yield richer data, but simpler web-based, screen-based, mobile, or hybrid approaches may be easier to implement across diverse settings, provided assessment is carefully designed [59]. Without implementation data on feasibility, acceptability, fidelity of use, sustainability, and cost, it remains difficult to judge whether a platform that performs well in a single study can support wider preparedness capacity across different health systems [11,59]. Positioning technology choices within health-system preparedness rather than simulation sophistication alone would make this literature more useful to policy and practice [10,11,60].

### 4.4. Clinical and public health practice implications

For clinical practice, the findings suggest that prehospital education programs should specify the tasks to be rehearsed, including triage prioritization, communication, and time-sensitive decision-making, before selecting a training platform. Where feasible, performance records should be linked to an explicit assessment standard and used in feedback and debriefing to identify learning needs. Embedded logs can support this process, but data capture alone should not be interpreted as evidence of better clinical performance. Applied simulation outcomes, including L2+, should be distinguished from evidence of transfer to routine practice or actual MCI response.

For public health practice, EMS leaders and preparedness planners should consider access, repeatability, infrastructure, technical support, and ongoing costs alongside instructional and assessment requirements. Screen-based, mobile, or hybrid approaches may warrant local evaluation where immersive systems are difficult to sustain. The absence of studies from low- or lower-middle-income countries means that feasibility, acceptability, and equitable access in these settings cannot be assumed from the present evidence. Training investments should therefore be accompanied by context-specific implementation evaluation [10,11,59,60].

These implications are priorities for training design and implementation, not recommendations based on demonstrated patient benefit. The review did not establish comparative effectiveness, and no included study reported organizational or patient-level outcomes. Future evaluations should assess whether improvements in simulated performance are sustained and translate into better clinical and system-level response.

### 4.5. A roadmap for future research and reporting

Across the corpus, the same reporting needs recurred often enough to warrant a single consolidated roadmap. Future studies should explicitly state what role each technology plays in the training architecture; specify which metrics are captured automatically, when capture occurs, and how those data inform feedback or debriefing; report the training design framework or learning theory used alongside the operational model being taught; prespecify whether embedded simulation performance is being interpreted as Level 2 or L2+ and state the assessment standard used; and compare equivalent MCI content across platforms that vary in immersion but use the same outcome definitions and follow-up structure [12,57]. Because implementation reach is central to disaster preparedness, future evaluations should also report feasibility, scalability, maintenance requirements, and equity-relevant constraints rather than only learner satisfaction or short-term knowledge change [11,59,60].

### 4.6. Limitations and strengths

This review has several limitations. The search was restricted to English-language studies published from 2015 onward, which may have excluded relevant earlier or non-English literature. As a scoping review, it did not include a formal risk-of-bias appraisal, so the findings should be interpreted as a map of the evidence base rather than a judgment of effectiveness certainty. The corpus was heterogeneous in populations, technologies, study designs, and outcome definitions, and it included three protocols without completed outcome data. In addition, our modified Kirkpatrick mapping, including the L2+ category and the five analytical constructs, involved interpretive judgment despite duplicate extraction and consensus processes. Finally, the review did not include stakeholder consultation, which may have limited practice-based insight into implementation priorities.

These limitations should be considered alongside important strengths. To our knowledge, this review is among the first broad scoping reviews to examine technology-enhanced prehospital MCI training across multiple modalities rather than focusing only on immersive technologies. Methodological strengths include prospectively registered methods, duplicate screening and extraction, explicit framework extraction, and structured outcome mapping using a modified Kirkpatrick model. Conceptually, the review offers a functional perspective by examining how technologies operate within training and assessment processes, helping explain differences in reported outcomes beyond simple modality labels.

## 5. Conclusions

As technology plays an increasingly central role in prehospital mass-casualty incident preparedness, advancing educational rigor requires a fundamental shift in how training platforms are evaluated and deployed. The findings of this review demonstrate that an educational tool’s value is defined less by its visual immersion or technological sophistication than by its underlying functional architecture. Dual-use platforms that seamlessly integrate scenario delivery with embedded, real-time performance logging consistently yield deeper, more defensible competency evaluations than systems prioritizing surface fidelity alone. Furthermore, closing the pervasive pedagogical transparency gap—where operational models are transparent but instructional designs remain opaque—is essential to ensuring that training interventions can be reliably replicated, compared, and synthesized across health systems.

These insights carry immediate, high-stakes implications for disaster risk reduction policy and resource allocation. Institutional procurement strategies for MCI preparedness must pivot from evaluating superficial visual capabilities toward prioritizing integrated measurement capacity and pedagogical alignment. Simultaneously, future research must actively dismantle the geographic and socioeconomic imbalances dominating the current literature. Addressing the scalability-rigor tension requires developing and testing cost-effective, low-barrier technological solutions—such as web-based or hybrid platforms with robust assessment logic—that can be sustainably implemented across low- and middle-income settings. By adopting standardized, framework-driven reporting that incorporates intermediate applied-performance metrics (such as the L2+ classification), the field can move beyond isolated, reaction-based evaluations and build an equitable, evidence-based global foundation for prehospital disaster preparedness.

## Supporting information

S1 File PRISMA-ScR Checklist. (DOCX)

S1 Appendix Full Electronic Search Strategies. (DOCX)

S2 Appendix Operational definitions and rules for study-level construct assignment. (DOCX)

S3 Appendix Technology Functional Classification of Included Studies. (DOCX)

S4 Appendix Data Collection Instruments, Data Types, and Assessment Timing. (DOCX)

## References

[1] World Health Organization. Mass casualty management systems: strategies and guidelines for building health sector capacity. Geneva: World Health Organization; 2007.

[2] DeNolf RL, Kahwaji CI. EMS mass casualty management. StatPearls. Treasure Island (FL): StatPearls Publishing; 2025. Available from: http://www.ncbi.nlm.nih.gov/books/NBK482373/29493995

[3] Agri J, Söderin L, Hammarberg E, Lennquist-Montán K, Montán C. Prehospital Preparedness for Major Incidents in Sweden: A National Survey with Focus on Mass-Casualty Incidents. Prehosp Disaster Med. 2023;38(1):65–72. doi: 10.1017/S1049023X22002229 36440661

[4] Hwang K il, Kim J. The Training Effects of Mass Casualty Triage in Radiological Events for 119 Emergency Medical Team. Prehosp Disaster med. 2023;38(S1):s129–s129. doi: 10.1017/s1049023x23003412

[5] Mills B, Dykstra P, Hansen S, Miles A, Rankin T, Hopper L, et al. Virtual Reality Triage Training Can Provide Comparable Simulation Efficacy for Paramedicine Students Compared to Live Simulation-Based Scenarios. Prehosp Emerg Care. 2020;24(4):525–36. doi: 10.1080/10903127.2019.1676345 31580178

[6] Heldring S, Jirwe M, Wihlborg J, Berg L, Lindström V. Using High-Fidelity Virtual Reality for Mass-Casualty Incident Training by First Responders - A Systematic Review of the Literature. Prehosp Disaster Med. 2024;39(1):94–105. doi: 10.1017/S1049023X24000049 38328887 PMC10882557

[7] Grimwood T, Snell L. The use of technology in healthcare education: a literature review. MedEdPublish. 2020;9:137. doi: 10.15694/mep.2020.000137.1

[8] Duan Y-Y, Zhang J-Y, Xie M, Feng X-B, Xu S, Ye Z-W. Erratum to: Application of Virtual Reality Technology in Disaster Medicine. Curr Med Sci. 2020;40(6):1205. doi: 10.1007/s11596-020-2278-x 33351165 PMC7801311

[9] Del Carmen Cardós-Alonso M, Otero-Varela L, Redondo M, Uzuriaga M, González M, Vazquez T, et al. Extended reality training for mass casualty incidents: a systematic review on effectiveness and experience of medical first responders. Int J Emerg Med. 2024;17(1):99. doi: 10.1186/s12245-024-00685-3 39179965 PMC11342566

[10] United Nations Office for Disaster Risk Reduction, Sendai Framework for Disaster Risk Reduction 2015–2030, UNDRR, Geneva. 2015. Available from: https://www.undrr.org/publication/sendai-framework-disaster-risk-reduction-2015-2030

[11] World Health Organization. Emergency response framework. 2nd edition. Geneva: WHO; 2024. Available from: https://www.who.int/publications/i/item/9789240058064

[12] Khalil H, Jia R, Moraes EB, Munn Z, Alexander L, Peters MDJ, et al. Scoping reviews and their role in identifying research priorities. J Clin Epidemiol. 2025;181:111712. doi: 10.1016/j.jclinepi.2025.111712 39924126

[13] Arksey H, O’Malley L. Scoping studies: towards a methodological framework. Int J Soc Res Methodol. 2005;8(1):19–32. doi: 10.1080/1364557032000119616

[14] Levac D, Colquhoun H, O’Brien KK. Scoping studies: advancing the methodology. Implement Sci. 2010;5:69. doi: 10.1186/1748-5908-5-69 20854677 PMC2954944

[15] Tricco AC, Lillie E, Zarin W, O’Brien KK, Colquhoun H, Levac D, et al. PRISMA Extension for Scoping Reviews (PRISMA-ScR): Checklist and Explanation. Ann Intern Med. 2018;169(7):467–73. doi: 10.7326/M18-0850 30178033

[16] Saouli A, Nour R, Farhan H, Omayer A, Yousif A, Hubloue I, et al. Tech-Based Training Approaches for Prehospital Mass Casualty Response: A Scoping Review Protocol, protocols.io. 2025. Available from: https://www.protocols.io/view/tech-based-training-approaches-for-prehospital-mas-g4pmbyvk7

[17] Saouli A, AlRahma A, Nour R, Farhan H, Omayer A, Yousif A, et al. Technology-enhanced training for prehospital mass-casualty response: a scoping review protocol. Preprints. 2025. doi: 10.20944/preprints202510.0634.v1 PMC13759121 43019319

[18] McGowan J, Sampson M, Salzwedel DM, Cogo E, Foerster V, Lefebvre C. PRESS Peer Review of Electronic Search Strategies: 2015 guideline statement. J Clin Epidemiol. 2016;75:40–6. doi: 10.1016/j.jclinepi.2016.01.021 27005575

[19] Covidence Systematic Review Software, Veritas Health Innovation, Melbourne, Australia. Available from: www.covidence.org

[20] Radianti J, Majchrzak TA, Fromm J, Wohlgenannt I. A systematic review of immersive virtual reality applications for higher education: design elements, lessons learned, and research agenda. Comput Educ. 2020;147:103778. doi: 10.1016/j.compedu.2019.103778

[21] Royal College of Physicians and Surgeons of Canada. CanMEDS 2015 Physician Competency Framework. Ottawa (ON): Royal College of Physicians and Surgeons of Canada; 2015.

[22] Falletta S. Evaluating training programs: the four levels. Am J Eval. 1998;19(2):259–61. doi: 10.1016/S1098-2140(99)80206-9

[23] Carnell S, Gomes De Siqueira A, Miles A, Lok B. Informing and Evaluating Educational Applications With the Kirkpatrick Model in Virtual Environments: Using a Virtual Human Scenario to Measure Communication Skills Behavior Change. Front Virtual Real. 2022;3. doi: 10.3389/frvir.2022.810797

[24] Voicescu GT, Lamine H, Loșonți AE, Lupan-Mureșan EM, Luka S, Ulerio JG, et al. Monitoring and evaluation in disaster management courses: a scoping review. BMC Med Educ. 2025;25(1):188. doi: 10.1186/s12909-025-06659-0 39910476 PMC11800627

[25] Cicero MX, Whitfill T, Munjal K, Madhok M, Diaz MCG, Scherzer DJ, et al. 60 seconds to survival: A pilot study of a disaster triage video game for prehospital providers. Am J Disaster Med. 2017;12(2):75–83. doi: 10.5055/ajdm.2017.0263 29136270

[26] Chumvanichaya K, Yuksen C, Nuanprom P, Aramvanitch K. A comparison of SIEVE, SORT, and START triage training effectiveness between immersive interactive 3D learning materials using virtual reality (VR-SSST) and traditional methods in mass casualty incidents. Int J Emerg Med. 2025;18(1):55. doi: 10.1186/s12245-025-00850-2 40082745 PMC11905642

[27] Heldring S, Jirwe M, Wihlborg J, Lindström V. Acceptability and applicability of using virtual reality for training mass casualty incidents- a mixed method study. BMC Med Educ. 2025;25(1):728. doi: 10.1186/s12909-025-07319-z 40389938 PMC12090617

[28] Wetherell MA, Williams G, Doran J. Assessing the psychobiological demands of high-fidelity training in pre-hospital emergency medicine. Scand J Trauma Resusc Emerg Med. 2024;32(1):101. doi: 10.1186/s13049-024-01272-4 39385220 PMC11462650

[29] ChiCTR2300072282. Virtual reality-based nursing training for mass casualty incidents, Chinese Clinical Trial Registry. 2023. Available from: https://www.chictr.org.cn/hvshowproject.html?id=228057&v=1.0

[30] Alhawatmeh HN, Rawashdeh SA, Alwidyan MT, Abuhammad S. Comparing virtual reality and live standardized patient drill simulation-based triage training methods in terms of triage knowledge and performance. Clin Simul Nurs. 2025;103:101749. doi: 10.1016/j.ecns.2025.101749

[31] Jain TN, Ragazzoni L, Stryhn H, Stratton SJ, Della Corte F. Comparison of the Sacco Triage Method Versus START Triage Using a Virtual Reality Scenario in Advance Care Paramedic Students. CJEM. 2016;18(4):288–92. doi: 10.1017/cem.2015.102 26553510

[32] Cicero MX, Whitfill T, Walsh B, Diaz MCG, Arteaga GM, Scherzer DJ, et al. Correlation Between Paramedic Disaster Triage Accuracy in Screen-Based Simulations and Immersive Simulations. Prehosp Emerg Care. 2019;23(1):83–9. doi: 10.1080/10903127.2018.1475530 30130424

[33] Masoumian Hosseini M, Masoumian Hosseini ST, Qayumi K, Hosseinzadeh S, Ahmady S. Crossover design in triage education: the effectiveness of simulated interactive vs. routine training on student nurses’ performance in a disaster situation. BMC Res Notes. 2023;16(1):313. doi: 10.1186/s13104-023-06596-5 37926836 PMC10626668

[34] Park SK, Kim HJ. Development and Evaluation of Virtual Reality-based Simulation Content for Nursing Students Regarding Emergency Triage. J Korean Acad Fundam Nurs. 2023;30(2):292–301. doi: 10.7739/jkafn.2022.30.2.292

[35] Chang C-W, Lin C-W, Huang C-Y, Hsu C-W, Sung H-Y, Cheng S-F. Effectiveness of the virtual reality chemical disaster training program in emergency nurses: A quasi experimental study. Nurse Educ Today. 2022;119:105613. doi: 10.1016/j.nedt.2022.105613 36327790

[36] NCT06034184. Enhancing mass casualty triage through virtual reality simulation, ClinicalTrials.gov. 2024. Available from: https://clinicaltrials.gov/study/NCT06034184

[37] Bajow N, Djalali A, Ingrassia PL, Ragazzoni L, Ageely H, Bani I, et al. Evaluation of a new community-based curriculum in disaster medicine for undergraduates. BMC Med Educ. 2016;16(1):225. doi: 10.1186/s12909-016-0746-6 27562428 PMC5000399

[38] Heldring S, Lindström V, Jirwe M, Wihlborg J. Exploring ambulance clinicians’ clinical reasoning when training mass casualty incidents using virtual reality: a qualitative study. Scand J Trauma Resusc Emerg Med. 2024;32(1):90. doi: 10.1186/s13049-024-01255-5 39285463 PMC11403774

[39] Lochmannová A. Exploring the role of virtual reality in preparing emergency responders for mass casualty incidents. Isr J Health Policy Res. 2025;14(1):22. doi: 10.1186/s13584-025-00681-9 40205512 PMC11984235

[40] McCoy CE, Alrabah R, Weichmann W, Langdorf MI, Ricks C, Chakravarthy B, et al. Feasibility of Telesimulation and Google Glass for Mass Casualty Triage Education and Training. West J Emerg Med. 2019;20(3):512–9. doi: 10.5811/westjem.2019.3.40805 31123554 PMC6526878

[41] Chevalier S, Paquay M, Goffoy J, Servotte JC, Stipulante S, Ghuysen A. Impact of virtual reality on performance among undergraduate healthcare professionals: a cross-sectional study. Int J Healthc Manag. 2025;18(2):185–94. doi: 10.1080/20479700.2023.2281066

[42] Foronda CL, Shubeck K, Swoboda SM, Hudson KW, Budhathoki C, Sullivan N, et al. Impact of virtual simulation to teach concepts of disaster triage. Clin Simul Nurs. 2016;12(4):137–44. doi: 10.1016/j.ecns.2016.02.004

[43] Way DP, Panchal AR, Price A, Berezina-Blackburn V, Patterson J, McGrath J, et al. Learner evaluation of an immersive virtual reality mass casualty incident simulator for triage training. BMC Digit Health. 2024;2(1):56. doi: 10.1186/s44247-024-00117-5 39290871 PMC11402856

[44] Baetzner AS, Hill Y, Roszipal B, Gerwann S, Beutel M, Birrenbach T, et al. Mass Casualty Incident Training in Immersive Virtual Reality: Quasi-Experimental Evaluation of Multimethod Performance Indicators. J Med Internet Res. 2025;27:e63241. doi: 10.2196/63241 39869892 PMC11811659

[45] Hosseini M, Masoumian Hosseini ST, Qayumi K. Nursing student satisfaction with a crisis management game-based training; a quasi-experimental study. Iran J Emerg Med. 2023;10(1):e22. doi: 10.22037/ijem.v10i1.42407 PMC9676700 36426168

[46] Hermann S, Gerstner J, Weiss F, Aichele S, Stricker E, Gorgati E, et al. Presentation and evaluation of a modern course in disaster medicine and humanitarian assistance for medical students. BMC Med Educ. 2021;21(1):610. doi: 10.1186/s12909-021-03043-6 34893083 PMC8661312

[47] Sibley AK, Jain TN, Butler M, Nicholson B, Sibley D, Smith D, et al. Remote Scene Size-up Using an Unmanned Aerial Vehicle in a Simulated Mass Casualty Incident. Prehosp Emerg Care. 2019;23(3):332–9. doi: 10.1080/10903127.2018.1511765 30122093

[48] Goldberg BS, Hall JE, Pham PK, Cho CS. Text messages by wireless mesh network vs voice by two-way radio in disaster simulations: A crossover randomized-controlled trial. Am J Emerg Med. 2021;48:148–55. doi: 10.1016/j.ajem.2021.04.004 33906052

[49] NCT06253156. The effect of virtual reality-based disaster education given to nursing students on disaster preparedness: randomized controlled study, ClinicalTrials.gov. 2024. Available from: https://clinicaltrials.gov/study/NCT06253156

[50] Bauchwitz B, Nguyen J, Woods K, Albagli K, Sawitz M, Hatch M, et al. The Use of Smartphone-Based Highly Realistic MCI Training as an Adjunct to Traditional Training Methods. Mil Med. 2024;189(Suppl 3):775–83. doi: 10.1093/milmed/usae274 39160830

[51] Shujuan L, Mawpin T, Meichan C, Weijun X, Jing W, Biru L. The Use of Virtual Reality to Improve Disaster Preparedness Among Nursing Students: A Randomized Study. J Nurs Educ. 2022;61(2):93–6. doi: 10.3928/01484834-20211213-05 35112954

[52] Hu H, Lai X, Yan L. Training nurses in an international emergency medical team using a serious role-playing game: a retrospective comparative analysis. BMC Med Educ. 2024;24(1):432. doi: 10.1186/s12909-024-05442-x 38649943 PMC11034038

[53] Pek JH, Quah LJJ, Valente M, Ragazzoni L, Della Corte F. Use of Simulation in Full-Scale Exercises for Response to Disasters and Mass-Casualty Incidents: A Scoping Review. Prehosp Disaster Med. 2023;38(6):792–806. doi: 10.1017/S1049023X2300660X 37997445

[54] Cook DA, Hamstra SJ, Brydges R, Zendejas B, Szostek JH, Wang AT, et al. Comparative effectiveness of instructional design features in simulation-based education: systematic review and meta-analysis. Med Teach. 2013;35(1):e867-98. doi: 10.3109/0142159X.2012.714886 22938677

[55] Costa JM, Miranda GL, Melo M. Four-component instructional design (4C/ID) model: a meta-analysis on use and effect. Learn Environ Res. 2022;25(2):445–63. doi: 10.1007/s10984-021-09373-y

[56] Clarkson L, Williams M. EMS mass casualty triage. In: StatPearls. Treasure Island (FL): StatPearls Publishing; 2025. Available from: http://www.ncbi.nlm.nih.gov/books/NBK459369/29083791

[57] Ebrahimi F, Masoudian T, Khiabani MM. Integrating ADDIE Needs Assessment with Kirkpatrick Evaluation: A Systematic Review. Asian J Educ Soc Stud. 2025;51(3):350–76. doi: 10.9734/ajess/2025/v51i31832

[58] Alliger GM, Tannenbaum SI, Bennett W, Traver H, Shotland A. A meta-analysis of the relations among training criteria. Pers Psychol. 1997;50(2):341–58. doi: 10.1111/j.1744-6570.1997.tb00911.x

[59] Ndibalema P. Constraints of transition to online distance learning in Higher Education Institutions during COVID-19 in developing countries: A systematic review. E-Learn Digit Media. 2022;19(6):595–618. doi: 10.1177/20427530221107510

[60] Nemani S. Barriers and Enablers to Adopting Virtual Reality in Lower Secondary STEAM Curricula. JARE. 2025;4(2):1–14. doi: 10.56397/jare.2025.03.01

