# Supplementary material for: Technology-enhanced training for prehospital mass-casualty incident preparedness: A scoping review
Source: PLoS One. 2026 Nov 12;21(11):e0348829. doi: 10.1371/journal.pone.0348829 (PMC13759121; doi:10.1371/journal.pone.0348829)
Supplement: Supplementary file 2 [file pone.0348829.s002.docx]

# Appendix S1. Full Electronic Search Strategies

Search strategies were developed on 13 June 2025 and peer-reviewed according to the PRESS (Peer Review of Electronic Search Strategies) guidelines. Searches combined keywords and controlled vocabulary (MeSH terms where applicable) across five conceptual blocks: (1) disaster/MCI context, (2) technology/concept, (3) population, (4) education/training, and (5) evaluation/outcomes. Database-specific adaptations preserved Boolean logic while conforming to each platform's syntax requirements.

**Search date:** 13 June 2025. Searches were updated prior to final analysis.

**PubMed**

("mass casualty incident" OR "Mass Casualty Incidents"[MeSH] OR "crisis" OR "Disasters"[Mesh] OR "aircraft accident" OR "avalanche" OR "biological accident" OR "biohazard release" OR "bioterrorism" OR "bomb" OR "chemical accident" OR "chemical hazard release" OR "chemical terrorism" OR "climate change" OR "cyclonic storm" OR "disaster victim" OR "disaster" OR "drought" OR "earthquake" OR "emergency patient" OR "emergency shelter" OR "flood" OR "landslide" OR "mass-casualty incident" OR "mass disaster" OR "multiple trauma" OR "natural disaster" OR "nuclear accident" OR "pandemic" OR "radioactive hazard release" OR "radiation accident" OR "structure collapse" OR "traffic accident" OR "terrorism" OR "tidal wave" OR "tornado" OR "wildfire" OR "catastrophe" OR "catastrophic accident" OR "CBRN" OR "CBRNe" OR "high-risk situations" OR "human-made disasters" OR "terrorist attack") AND ("eye-tracking" OR "Technology"[Mesh] OR "virtual reality" OR "augmented reality" OR "mixed reality" OR "biometric sensors" OR "electroencephalogram" OR "digital monitoring" OR "motion tracking" OR "wearable devices" OR "performance dashboards" OR "psychophysiological tools" OR "galvanic skin response" OR "pupillometry" OR "heart rate variability" OR "Eye-Tracking Technology"[Mesh] OR "Virtual Reality"[Mesh] OR "Augmented Reality"[Mesh] OR "Spectroscopy, Near-Infrared"[Mesh] OR "Galvanic Skin Response"[Mesh] OR "artificial intelligence" OR "augmented reality" OR "human computer interaction" OR "human machine interface" OR "microcomputer" OR "mobile application" OR "user-centered design" OR "video game" OR "virtual reality" OR "wearable computer" OR "ambient intelligence" OR "audiovisual aids" OR "data display" OR "game theory" OR "human-computer interaction" OR "medical informatics applications" OR "smartphone" OR "smart glasses" OR "user-computer interface" OR "video games" OR "wearable electronic devices" OR "3D environment" OR "3-D environment" OR "AI" OR "augmented virtuality" OR "close-to-reality" OR "computer environment" OR "digital tools" OR "enhanced realism" OR "enhanced reality" OR "extended reality" OR "game-based" OR "gamification" OR "haptic-based" OR "haptic technology" OR "immersive virtual reality" OR "immersive virtual environment" OR "realistic scenarios" OR "real-world scenarios" OR "smart electronic devices" OR "smart wearables" OR "virtual patient" OR "virtual scenarios" OR "virtual worlds" OR "VR" OR "wearable technologies" OR "Wearable Proximity Sensors" OR Wearable OR GPS OR "GPS-Based" OR "High-Fidelity" OR "Serious games") AND ("first responders" OR "medical intern" OR "healthcare provider" OR "Paramedics"[MeSH] OR "Health Personnel"[MeSH] OR "Nurses"[MeSH] OR "Physicians"[MeSH]) AND ("Education"[Mesh] OR "Simulation Training"[Mesh] OR Training OR "Feedback training" OR "Simulation-based education" OR "Real-world training" OR "Interprofessional Education" OR "Competency-Based Education") AND (evaluate OR assess OR train OR outcomes)

**Scopus**

(TITLE-ABS-KEY("mass casualty incident" OR "crisis" OR "Disasters" OR "aircraft accident" OR "avalanche" OR "biological accident" OR "biohazard release" OR "bioterrorism" OR "bomb" OR "chemical accident" OR "chemical hazard release" OR "chemical terrorism" OR "cyclonic storm" OR "disaster" OR "drought" OR "earthquake" OR "emergency shelter" OR "flood" OR "landslide" OR "mass-casualty incident" OR "mass disaster" OR "multiple trauma" OR "natural disaster" OR "nuclear accident" OR "pandemic" OR "radioactive hazard release" OR "radiation accident" OR "structure collapse" OR "traffic accident" OR "terrorism" OR "tidal wave" OR "tornado" OR "wildfire" OR "catastrophe" OR "catastrophic accident" OR "CBRN" OR "CBRNe" OR "high-risk situations" OR "human-made disasters" OR "terrorist attack")) AND (TITLE-ABS-KEY("eye-tracking" OR "Technology" OR "virtual reality" OR "augmented reality" OR "mixed reality" OR "biometric sensors" OR "electroencephalogram" OR "digital monitoring" OR "motion tracking" OR "wearable devices" OR "performance dashboards" OR "psychophysiological tools" OR "galvanic skin response" OR "pupillometry" OR "heart rate variability" OR "artificial intelligence" OR "human computer interaction" OR "human machine interface" OR "microcomputer" OR "mobile application" OR "user-centered design" OR "video game" OR "wearable computer" OR "ambient intelligence" OR "audiovisual aids" OR "data display" OR "game theory" OR "human-computer interaction" OR "medical informatics applications" OR "smartphone" OR "smart glasses" OR "user-computer interface" OR "video games" OR "wearable electronic devices" OR "3D environment" OR "AI" OR "augmented virtuality" OR "close-to-reality" OR "computer environment" OR "digital tools" OR "enhanced realism" OR "enhanced reality" OR "extended reality" OR "game-based" OR "gamification" OR "haptic-based" OR "haptic technology" OR "immersive virtual reality" OR "immersive virtual environment" OR "realistic scenarios" OR "real-world scenarios" OR "smart electronic devices" OR "smart wearables" OR "virtual patient" OR "virtual scenarios" OR "virtual worlds" OR "VR" OR "wearable technologies" OR "Wearable Proximity Sensors" OR Wearable OR GPS OR "GPS-Based" OR "High-Fidelity" OR "Serious games")) AND (TITLE-ABS-KEY("first responders" OR "medical intern" OR "healthcare provider" OR "Paramedics" OR "Health Personnel" OR "Nurses" OR "Physicians")) AND (TITLE-ABS-KEY("Education" OR "Simulation Training" OR Training OR "Feedback training" OR "Simulation-based education" OR "Real-world training" OR "Interprofessional Education" OR "Competency-Based Education")) AND (TITLE-ABS-KEY("evaluate" OR "assess" OR "train" OR "outcomes")) AND (LIMIT-TO(SUBJAREA, "MEDI") OR LIMIT-TO(SUBJAREA, "NURS") OR LIMIT-TO(SUBJAREA, "HEAL") OR LIMIT-TO(SUBJAREA, "NEUR") OR LIMIT-TO(SUBJAREA, "MULT"))

**Embase**

#1: 'mass casualty incident' OR 'crisis' OR 'disasters' OR 'aircraft accident' OR 'avalanche' OR 'biological accident' OR 'biohazard release' OR 'bioterrorism' OR 'bomb' OR 'chemical accident' OR 'chemical hazard release' OR 'chemical terrorism' OR 'cyclonic storm' OR 'disaster' OR 'drought' OR 'earthquake' OR 'flood' OR 'landslide' OR 'mass-casualty incident' OR 'mass disaster' OR 'multiple trauma' OR 'natural disaster' OR 'nuclear accident' OR 'pandemic' OR 'radioactive hazard release' OR 'radiation accident' OR 'structure collapse' OR 'traffic accident' OR 'terrorism' OR 'tidal wave' OR 'tornado' OR 'wildfire' OR 'catastrophe' OR 'catastrophic accident' OR 'cbrn' OR 'cbrne' OR 'high-risk situations' OR 'human-made disasters' OR 'terrorist attack' #2: 'eye-tracking' OR 'technology' OR 'mixed reality' OR 'biometric sensors' OR 'electroencephalogram' OR 'digital monitoring' OR 'motion tracking' OR 'wearable devices' OR 'performance dashboards' OR 'psychophysiological tools' OR 'pupillometry' OR 'heart rate variability' OR 'artificial intelligence' OR 'augmented reality' OR 'human computer interaction' OR 'human machine interface' OR 'microcomputer' OR 'mobile application' OR 'user-centered design' OR 'video game' OR 'virtual reality' OR 'wearable computer' OR 'ambient intelligence' OR 'audiovisual aids' OR 'data display' OR 'game theory' OR 'human-computer interaction' OR 'medical informatics applications' OR 'smartphone' OR 'smart glasses' OR 'user-computer interface' OR 'video games' OR 'wearable electronic devices' OR '3d environment' OR 'ai' OR 'augmented virtuality' OR 'close-to-reality' OR 'computer environment' OR 'digital tools' OR 'enhanced realism' OR 'enhanced reality' OR 'extended reality' OR 'game-based' OR 'gamification' OR 'haptic-based' OR 'haptic technology' OR 'immersive virtual reality' OR 'immersive virtual environment' OR 'realistic scenarios' OR 'real-world scenarios' OR 'smart electronic devices' OR 'smart wearables' OR 'virtual patient' OR 'virtual scenarios' OR 'virtual worlds' OR 'vr' OR 'wearable technologies' OR 'wearable proximity sensors' OR 'wearable' OR 'gps' OR 'gps-based' OR 'high-fidelity' OR 'serious games' #3: 'first responders' OR 'medical intern' OR 'healthcare provider' OR 'paramedics' OR 'health personnel' OR 'nurses' OR 'physicians' #4: 'education' OR 'simulation training' OR training OR 'feedback training' OR 'simulation-based education' OR 'real-world training' OR 'interprofessional education' OR 'competency-based education' #5: 'evaluate' OR 'assess' OR 'train' OR 'outcomes' #6: #1 AND #2 AND #3 AND #4 AND #5

**CINAHL**

("mass casualty incident" OR "crisis" OR "Disasters" OR "aircraft accident" OR "avalanche" OR "biological accident" OR "biohazard release" OR "bioterrorism" OR "bomb" OR "chemical accident" OR "chemical hazard release" OR "chemical terrorism" OR "cyclonic storm" OR "disaster" OR "drought" OR "earthquake" OR "emergency shelter" OR "flood" OR "landslide" OR "mass-casualty incident" OR "mass disaster" OR "multiple trauma" OR "natural disaster" OR "nuclear accident" OR "pandemic" OR "radioactive hazard release" OR "radiation accident" OR "structure collapse" OR "traffic accident" OR "terrorism" OR "tidal wave" OR "tornado" OR "wildfire" OR "catastrophe" OR "catastrophic accident" OR "CBRN" OR "CBRNe" OR "high-risk situations" OR "human-made disasters" OR "terrorist attack") AND ("eye-tracking" OR "Technology" OR "virtual reality" OR "augmented reality" OR "mixed reality" OR "biometric sensors" OR "electroencephalogram" OR "digital monitoring" OR "motion tracking" OR "wearable devices" OR "performance dashboards" OR "psychophysiological tools" OR "galvanic skin response" OR "pupillometry" OR "heart rate variability" OR "artificial intelligence" OR "human computer interaction" OR "human machine interface" OR "microcomputer" OR "mobile application" OR "user-centered design" OR "video game" OR "wearable computer" OR "ambient intelligence" OR "audiovisual aids" OR "data display" OR "game theory" OR "human-computer interaction" OR "medical informatics applications" OR "smartphone" OR "smart glasses" OR "user-computer interface" OR "video games" OR "wearable electronic devices" OR "3D environment" OR "AI" OR "augmented virtuality" OR "close-to-reality" OR "computer environment" OR "digital tools" OR "enhanced realism" OR "enhanced reality" OR "extended reality" OR "game-based" OR "gamification" OR "haptic-based" OR "haptic technology" OR "immersive virtual reality" OR "immersive virtual environment" OR "realistic scenarios" OR "real-world scenarios" OR "smart electronic devices" OR "smart wearables" OR "virtual patient" OR "virtual scenarios" OR "virtual worlds" OR "VR" OR "wearable technologies" OR "Wearable Proximity Sensors" OR Wearable OR GPS OR "GPS-Based" OR "High-Fidelity" OR "Serious games") AND ("first responders" OR "medical intern" OR "healthcare provider" OR "Paramedics" OR "Health Personnel" OR "Nurses" OR "Physicians") AND ("Education" OR "Simulation Training" OR Training OR "Feedback training" OR "Simulation-based education" OR "Real-world training" OR "Interprofessional Education" OR "Competency-Based Education") AND ("evaluate" OR "assess" OR "train" OR "outcomes")

**PsycINFO (via APA PsycNet)**

((Any Field: ("mass casualty incident") OR Any Field: ("crisis") OR Any Field: ("Disasters") OR Any Field: ("aircraft accident") OR Any Field: ("avalanche") OR Any Field: ("biological accident") OR Any Field: ("biohazard release") OR Any Field: ("bioterrorism") OR Any Field: ("bomb") OR Any Field: ("chemical accident") OR Any Field: ("chemical hazard release") OR Any Field: ("chemical terrorism") OR Any Field: ("cyclonic storm") OR Any Field: ("disaster") OR Any Field: ("drought") OR Any Field: ("earthquake") OR Any Field: ("emergency shelter") OR Any Field: ("flood") OR Any Field: ("landslide") OR Any Field: ("mass-casualty incident") OR Any Field: ("mass disaster") OR Any Field: ("multiple trauma") OR Any Field: ("natural disaster") OR Any Field: ("nuclear accident") OR Any Field: ("pandemic") OR Any Field: ("radioactive hazard release") OR Any Field: ("radiation accident") OR Any Field: ("structure collapse") OR Any Field: ("traffic accident") OR Any Field: ("terrorism") OR Any Field: ("tidal wave") OR Any Field: ("tornado") OR Any Field: ("wildfire") OR Any Field: ("catastrophe") OR Any Field: ("catastrophic accident") OR Any Field: ("CBRN") OR Any Field: ("CBRNe") OR Any Field: ("high-risk situations") OR Any Field: ("human-made disasters") OR Any Field: ("terrorist attack"))) AND ((Any Field: ("eye-tracking") OR Any Field: ("Technology") OR Any Field: ("virtual reality") OR Any Field: ("augmented reality") OR Any Field: ("mixed reality") OR Any Field: ("biometric sensors") OR Any Field: ("electroencephalogram") OR Any Field: ("digital monitoring") OR Any Field: ("motion tracking") OR Any Field: ("wearable devices") OR Any Field: ("performance dashboards") OR Any Field: ("psychophysiological tools") OR Any Field: ("galvanic skin response") OR Any Field: ("pupillometry") OR Any Field: ("heart rate variability") OR Any Field: ("artificial intelligence") OR Any Field: ("human computer interaction") OR Any Field: ("human machine interface") OR Any Field: ("microcomputer") OR Any Field: ("mobile application") OR Any Field: ("user-centered design") OR Any Field: ("video game") OR Any Field: ("wearable computer") OR Any Field: ("ambient intelligence") OR Any Field: ("audiovisual aids") OR Any Field: ("data display") OR Any Field: ("game theory") OR Any Field: ("human-computer interaction") OR Any Field: ("medical informatics applications") OR Any Field: ("smartphone") OR Any Field: ("smart glasses") OR Any Field: ("user-computer interface") OR Any Field: ("video games") OR Any Field: ("wearable electronic devices") OR Any Field: ("3D environment") OR Any Field: ("AI") OR Any Field: ("augmented virtuality") OR Any Field: ("close-to-reality") OR Any Field: ("computer environment") OR Any Field: ("digital tools") OR Any Field: ("enhanced realism") OR Any Field: ("enhanced reality") OR Any Field: ("extended reality") OR Any Field: ("game-based") OR Any Field: ("gamification") OR Any Field: ("haptic-based") OR Any Field: ("haptic technology") OR Any Field: ("immersive virtual reality") OR Any Field: ("immersive virtual environment") OR Any Field: ("realistic scenarios") OR Any Field: ("real-world scenarios") OR Any Field: ("smart electronic devices") OR Any Field: ("smart wearables") OR Any Field: ("virtual patient") OR Any Field: ("virtual scenarios") OR Any Field: ("virtual worlds") OR Any Field: ("VR") OR Any Field: ("wearable technologies") OR Any Field: ("Wearable Proximity Sensors") OR Any Field: (Wearable) OR Any Field: (GPS) OR Any Field: ("GPS-Based") OR Any Field: ("High-Fidelity") OR Any Field: ("Serious games"))) AND ((Any Field: ("first responders") OR Any Field: ("medical intern") OR Any Field: ("healthcare provider") OR Any Field: ("Paramedics") OR Any Field: ("Health Personnel") OR Any Field: ("Nurses") OR Any Field: ("Physicians"))) AND ((Any Field: ("Education") OR Any Field: ("Simulation Training") OR Any Field: (Training) OR Any Field: ("Feedback training") OR Any Field: ("Simulation-based education") OR Any Field: ("Real-world training") OR Any Field: ("Interprofessional Education") OR Any Field: ("Competency-Based Education"))) AND ((Any Field: ("evaluate") OR Any Field: ("assess") OR Any Field: ("train") OR Any Field: ("outcomes")))

**Cochrane Library**

("mass casualty incident" OR "crisis" OR "Disasters" OR "aircraft accident" OR "avalanche" OR "biological accident" OR "biohazard release" OR "bioterrorism" OR "bomb" OR "chemical accident" OR "chemical hazard release" OR "chemical terrorism" OR "cyclonic storm" OR "disaster" OR "drought" OR "earthquake" OR "emergency shelter" OR "flood" OR "landslide" OR "mass-casualty incident" OR "mass disaster" OR "multiple trauma" OR "natural disaster" OR "nuclear accident" OR "pandemic" OR "radioactive hazard release" OR "radiation accident" OR "structure collapse" OR "traffic accident" OR "terrorism" OR "tidal wave" OR "tornado" OR "wildfire" OR "catastrophe" OR "catastrophic accident" OR "CBRN" OR "CBRNe" OR "high-risk situations" OR "human-made disasters" OR "terrorist attack"):ti,ab,kw AND ("eye-tracking" OR "Technology" OR "virtual reality" OR "augmented reality" OR "mixed reality" OR "biometric sensors" OR "electroencephalogram" OR "digital monitoring" OR "motion tracking" OR "wearable devices" OR "performance dashboards" OR "psychophysiological tools" OR "galvanic skin response" OR "pupillometry" OR "heart rate variability" OR "artificial intelligence" OR "human computer interaction" OR "human machine interface" OR "microcomputer" OR "mobile application" OR "user-centered design" OR "video game" OR "wearable computer" OR "ambient intelligence" OR "audiovisual aids" OR "data display" OR "game theory" OR "human-computer interaction" OR "medical informatics applications" OR "smartphone" OR "smart glasses" OR "user-computer interface" OR "video games" OR "wearable electronic devices" OR "3D environment" OR "AI" OR "augmented virtuality" OR "close-to-reality" OR "computer environment" OR "digital tools" OR "enhanced realism" OR "enhanced reality" OR "extended reality" OR "game-based" OR "gamification" OR "haptic-based" OR "haptic technology" OR "immersive virtual reality" OR "immersive virtual environment" OR "realistic scenarios" OR "real-world scenarios" OR "smart electronic devices" OR "smart wearables" OR "virtual patient" OR "virtual scenarios" OR "virtual worlds" OR "VR" OR "wearable technologies" OR "Wearable Proximity Sensors" OR Wearable OR GPS OR "GPS-Based" OR "High-Fidelity" OR "Serious games"):ti,ab,kw AND ("first responders" OR "medical intern" OR "healthcare provider" OR "Paramedics" OR "Health Personnel" OR "Nurses" OR "Physicians"):ti,ab,kw AND ("Education" OR "Simulation Training" OR Training OR "Feedback training" OR "Simulation-based education" OR "Real-world training" OR "Interprofessional Education" OR "Competency-Based Education"):ti,ab,kw AND ("evaluate" OR "assess" OR "train" OR "outcomes"):ti,ab,kw

**ClinicalTrials.gov**

("mass casualty incident" OR "crisis" OR "Disasters" OR "aircraft accident" OR "avalanche" OR "biological accident" OR "biohazard release" OR "bioterrorism" OR "bomb" OR "chemical accident" OR "chemical hazard release" OR "chemical terrorism" OR "cyclonic storm" OR "disaster" OR "drought" OR "earthquake" OR "emergency shelter" OR "flood" OR "landslide" OR "mass-casualty incident" OR "mass disaster" OR "multiple trauma" OR "natural disaster" OR "nuclear accident" OR "pandemic" OR "radioactive hazard release" OR "radiation accident" OR "structure collapse" OR "traffic accident" OR "terrorism" OR "tidal wave" OR "tornado" OR "wildfire" OR "catastrophe" OR "catastrophic accident" OR "CBRN" OR "CBRNe" OR "high-risk situations" OR "human-made disasters" OR "terrorist attack") AND ("eye-tracking" OR "Technology" OR "virtual reality" OR "augmented reality" OR "mixed reality" OR "biometric sensors" OR "electroencephalogram" OR "digital monitoring" OR "motion tracking" OR "wearable devices" OR "performance dashboards" OR "psychophysiological tools" OR "galvanic skin response" OR "pupillometry" OR "heart rate variability" OR "artificial intelligence" OR "augmented reality" OR "human computer interaction" OR "human machine interface" OR "microcomputer" OR "mobile application" OR "user-centered design" OR "video game" OR "virtual reality" OR "wearable computer" OR "ambient intelligence" OR "audiovisual aids" OR "data display" OR "game theory" OR "human-computer interaction" OR "medical informatics applications" OR "smartphone" OR "smart glasses" OR "user-computer interface" OR "video games" OR "wearable electronic devices" OR "3D environment" OR "AI" OR "augmented virtuality" OR "close-to-reality" OR "computer environment" OR "digital tools" OR "enhanced realism" OR "enhanced reality" OR "extended reality" OR "game-based" OR "gamification" OR "haptic-based" OR "haptic technology" OR "immersive virtual reality" OR "immersive virtual environment" OR "realistic scenarios" OR "real-world scenarios" OR "smart electronic devices" OR "smart wearables" OR "virtual patient" OR "virtual scenarios" OR "virtual worlds" OR "VR" OR "wearable technologies" OR "Wearable Proximity Sensors" OR Wearable OR GPS OR "GPS-Based" OR "High-Fidelity" OR "Serious games") AND ("first responders" OR "medical intern" OR "healthcare provider" OR "Paramedics" OR "Health Personnel" OR "Nurses" OR "Physicians") AND ("Education" OR "Simulation Training" OR Training OR "Feedback training" OR "Simulation-based education" OR "Real-world training" OR "Interprofessional Education" OR "Competency-Based Education") AND ("evaluate" OR "assess" OR "train" OR "outcomes")

**Supplementary sources:** Google Scholar and reference lists of included articles and relevant systematic reviews were manually screened to identify additional eligible records.
