# Supplementary material for: Technology-enhanced training for prehospital mass-casualty incident preparedness: A scoping review
Source: PLoS One. 2026 Nov 12;21(11):e0348829. doi: 10.1371/journal.pone.0348829 (PMC13759121; doi:10.1371/journal.pone.0348829)
Supplement: Supplementary file 3 [file pone.0348829.s003.docx]

**Appendix S2. Operational definitions and rules for study-level construct assignment**

| Construct | Construct type | Operational definition | Permitted categories | Required evidence | Decision rule |
| --- | --- | --- | --- | --- | --- |
| Technology Function Spectrum | Corpus-derived descriptive construct | The primary educational role played by the technology in the intervention as reported in the study | Delivery-only; Dual-use; Assessment-dedicated; Hybrid | Technology modality; stated purpose of technology; whether the same platform generated objective evaluative evidence used in the paper; whether functions were distributed across coordinated technologies | **Delivery-only**: technology primarily delivers content, scenario exposure, or simulation, while evaluative evidence is generated outside the platform. **Dual-use**: the same platform both delivers the training experience and generates objective evaluative evidence materially used in the study. **Assessment-dedicated**: technology is used primarily to measure performance, physiology, workload, or scene assessment rather than to deliver the main instructional experience. **Hybrid**: delivery and assessment functions are distributed across multiple coordinated technologies, or no single platform adequately represents the intervention as a whole. |
| Data Capture Architecture | Corpus-derived descriptive construct | The principal way outcome data were generated in relation to the training technology | Embedded; External; Multimodal | Outcome measures; assessment timing; instruments/tools; whether primary evidence came from system logs, in-platform metrics, observer ratings, tests, interviews, audio/video coding, or physiological recordings | **Embedded**: primary evaluative evidence is generated natively within the training platform during learner interaction. **External**: primary evaluative evidence comes from tools administered or analyzed outside the training platform, including observer ratings, checklists, interviews, questionnaires, knowledge tests, audio/video coding, and standalone physiological measures. **Multimodal**: both embedded and external sources contribute materially to the study’s evaluative claims. Ancillary satisfaction surveys alone do not convert an otherwise embedded study into multimodal. |
| Pedagogical Transparency Gap | Corpus-derived descriptive construct | The clarity with which the study reports and aligns its training design, assessment approach, and operational framework | High transparency; Partial transparency; Low transparency | Separate coding of the training design domain, assessment domain, and operational domain, followed by review of whether a linkage between what was taught and how it was assessed is explicitly identifiable | **High transparency**: all three domains are explicitly reported and at least one explicit linkage is provided between training objectives/content and assessment. **Partial transparency**: any two domains are explicitly reported, or all three domains are present but their alignment is unclear. **Low transparency**: zero or one domain is explicitly reported, or domains are implied rather than named or clearly described. |
