# Supplementary material for: Technology-enhanced training for prehospital mass-casualty incident preparedness: A scoping review
Source: PLoS One. 2026 Nov 12;21(11):e0348829. doi: 10.1371/journal.pone.0348829 (PMC13759121; doi:10.1371/journal.pone.0348829)
Supplement: Supplementary file 4 [file pone.0348829.s004.docx]

# Appendix S3. Technology Functional Classification of Included Studies

Each included study was classified along two analytical dimensions derived from the corpus: the Technology Function Spectrum (delivery-only, dual-use, assessment-dedicated, or hybrid) and the Data Capture Architecture (embedded, external, or multimodal). These constructs characterise how technology was used beyond simple modality classification and how outcome data were captured relative to the training platform. See main text for full definitions.

| **Study ID** | **Technology Modality** | **Specific Technology** | **Purpose** | **Function Spectrum** | **Data Capture** |
| --- | --- | --- | --- | --- | --- |
| Goldberg 2021 | Communication device | goTenna Mesh wireless mesh network; two-way radios | Training | Hybrid | Multimodal |
| McCoy 2019 | Telesimulation / Smart glasses | Google Glass with EyeSight; join.me; HF mannequins | Both | Hybrid | Multimodal |
| Cicero 2017 | Serious game | 60 Seconds to Survival (video game) | Training | Dual-use | Embedded |
| ChiCTR2300072282 | Virtual reality | VR-based nursing training system | Training | Delivery-only | External |
| Chumvanichaya 2025 | Virtual reality | VR-SSST immersive 3D learning materials | Training | Dual-use | Embedded |
| Hosseini 2023 (2) | Serious game | Crisis management game-based training | Training | Delivery-only | External |
| Way 2024 | Virtual reality | MCI-VR system; Meta Quest 2 HMD | Both | Dual-use | Embedded |
| Heldring 2025 | Virtual reality | GoSaveThem VR; HTC VIVE HMD; haptic controllers | Both | Dual-use | Embedded |
| Shujuan 2022 | Virtual reality | VR disaster simulation system | Both | Dual-use | Multimodal |
| Jain 2016 | Screen-based simulation | XVR Simulation System (E-Semble) | Training | Dual-use | Embedded |
| Cicero 2019 | Screen-based / immersive simulation | 60 Seconds to Survival game + live immersive simulation | Training | Dual-use | Embedded |
| Baetzner 2025 | Virtual reality + eye-tracking | XVR + Varjo Aero HMD with eye-tracking (200 Hz) | Both | Dual-use | Multimodal |
| Hermann 2021 | Computer-based simulation | tuevalon computer-based simulation platform | Both | Delivery-only | External |
| Kyoung 2023 | Virtual reality | VR-based emergency triage simulation | Training | Delivery-only | External |
| Hosseini 2023 (1) | Screen-based simulation | Simulated Interactive Guideline (SIG); Articulate Storyline 3 | Both | Dual-use | Embedded |
| NCT06253156 | Virtual reality | VR-based disaster education system | Training | Delivery-only | External |
| NCT06034184 | Virtual reality | VR mass casualty triage simulation | Training | Delivery-only | External |
| Wetherell 2024 | Wearable biometric | Garmin Vivosport/Vivosmart 5 smartwatches; salivary cortisol | Assessment | Assessment-dedicated | Multimodal |
| Hu 2024 | Serious game | Computer game simulation for IEMT training | Both | Dual-use | Embedded |
| Alhawatmeh 2025 | Virtual reality | Full-immersion VR triage simulation | Training | Delivery-only | External |
| Bauchwitz 2024 | Smartphone simulation | EFECTIVE smartphone-based training simulation | Training | Dual-use | Embedded |
| Chevalier 2023 | Virtual reality | VR START triage simulation | Training | Dual-use | Embedded |
| Heldring 2024 | Virtual reality | GoSaveThem VR; HTC VIVE HMD | Both | Dual-use | Embedded |
| Lochmannova 2025 | VR + wearable biometric | VR system + Garmin smartwatches (biometrics) | Both | Hybrid | Multimodal |
| Sibley 2018 | Unmanned aerial vehicle | UAV/drone for remote scene size-up | Assessment | Assessment-dedicated | External |
| Chang 2022 | Virtual reality | 360-degree VR simulation; HTC VIRTI platform | Training | Dual-use | Embedded |
| Foronda 2016 | Screen-based simulation | V-CAEST web-based 3D virtual simulation | Training | Dual-use | Embedded |
| Bajow 2016 | Multiple modalities | XVR VR simulation; ISEE; video lectures; e-learning | Training | Hybrid | External |

***Function Spectrum categories:*** *Delivery-only = content transmission without embedded assessment; Dual-use = simultaneous training and data capture; Assessment-dedicated = primarily designed for performance measurement; Hybrid = multi-modal systems combining elements across categories.*

***Data Capture categories:*** *Embedded = automatic logging within the training platform; External = separate assessment instruments administered independently; Multimodal = integration of embedded platform data with physiological measures or external assessments.*
