# Supplementary material for: Technology-enhanced training for prehospital mass-casualty incident preparedness: A scoping review
Source: PLoS One. 2026 Nov 12;21(11):e0348829. doi: 10.1371/journal.pone.0348829 (PMC13759121; doi:10.1371/journal.pone.0348829)
Supplement: Supplementary file 5 [file pone.0348829.s005.docx]

# Appendix S4. Data Collection Instruments, Data Types, and Assessment Timing

This appendix documents the specific instruments, tools, and data sources used for outcome measurement in each included study, together with the type of data collected and the timing of assessment relative to the training intervention. Studies are listed in the same order as the main synthesis.

| **Study ID** | **Data Type** | **Instruments / Tools** | **Assessment Timing** |
| --- | --- | --- | --- |
| Goldberg 2021 | Mixed | NASA Task Load Index (NASA-TLX); System Usability Scale (SUS); demographic questionnaire; audio recordings; smartphone screen captures; predefined answer key/coding protocol | During simulation (performance data); immediately post-simulation (NASA-TLX, SUS, demographics); end-of-study (device preference) |
| McCoy 2019 | Mixed | Five-point Likert-scale survey; triage accuracy assessment form; Google Glass with EyeSight software (real-time data capture) | During telesimulation (triage data); immediately post-course (survey responses) |
| Cicero 2017 | Quantitative | Game performance scores / video game logs (automated) | During simulation |
| ChiCTR2300072282 | Quantitative | Self-report questionnaire | Post-intervention |
| Chumvanichaya 2025 | Mixed | ARCS Motivation Survey; pre-/post-tests; standardised simulation evaluation; performance metrics (SORT triage protocol, GCS evaluation, correctness/time) | Pre, post, and 1-week follow-up |
| Hosseini 2023 (2) | Quantitative | Satisfaction questionnaire; State-Trait Anxiety Inventory (STAI) | Post-intervention |
| Way 2024 | Mixed | Custom post-encounter survey; open-ended questions | Post-simulation |
| Heldring 2025 | Mixed | Questionnaire (Likert items + open comments); interview protocol; VR computer recordings (time and accuracy) | During and post simulation |
| Shujuan 2022 | Quantitative | Disaster Preparedness Questionnaire (DPQ); self-developed confidence cards | Pre and post training |
| Jain 2016 | Quantitative | Stopwatch timing; evacuation order record | During simulation |
| Cicero 2019 | Quantitative | SBS (60 Seconds to Survival) game database logs: time, triage order, assessment actions, triage decisions (automated) | During simulation |
| Baetzner 2025 | Mixed | Structured web questionnaire; Varjo Aero eye-tracking (200 Hz); VR system performance logs (accuracy, speed); audio recordings | During and post simulation |
| Hermann 2021 | Quantitative | Pre-/post-course self-assessment survey; MCQ knowledge test; institutional survey (SurveyMonkey) | Pre and post intervention |
| Kyoung 2023 | Quantitative | Demographic survey (8 items); VR Usability Evaluation Tool (ARCS-based, 45 items) | Post-training |
| Hosseini 2023 (1) | Quantitative | Triage Knowledge Questionnaire (TKQ); Triage Skills Questionnaire (TSQ); Objective Structured Clinical Examination (OSCE); ICT Aptitude Survey | Pre and post training |
| NCT06253156 | Quantitative | Disaster Threat Preparedness and Psychological Preparedness Scale (planned) | Post-training (planned) |
| NCT06034184 | Quantitative | 20-item multiple-choice knowledge test (planned) | Post-training (planned) |
| Wetherell 2024 | Quantitative | State-Trait Anxiety Inventory (SF-SA); Competitive State Anxiety Inventory-2 (CSAI-2); Daily Anticipated vs. Experienced Demands Scale; Garmin smartwatches (HR); salivary cortisol (Salivettes) | Pre and post simulation |
| Hu 2024 | Quantitative | Training records database; cognitive load questionnaire; technology acceptance questionnaire; pre-/post-/final tests; learning motivation questionnaire | Pre, during, and post training |
| Alhawatmeh 2025 | Quantitative | Pre-/post-tests; START-Assessment-Global scale (5-point); 25-item triage knowledge test | Pre and post training |
| Bauchwitz 2024 | Mixed | Post-simulation perception survey (5-point Likert + open-ended responses) | Post-simulation |
| Chevalier 2023 | Mixed | Theoretical questionnaire (START); socio-professional survey; ITQF (Immersive Tendencies); observation (triage tag + timing); Mental Readiness Form; WCC; REI; Simulator Sickness Questionnaire; Sense of Presence questionnaire; self-report scale; MCQ | Pre, during, and post simulation |
| Heldring 2024 | Qualitative | Chart-Stimulated Recall (CSR) interviews; video recordings of VR session | Post-simulation |
| Lochmannova 2025 | Quantitative | VR system logs (triage accuracy, timing, critical errors); METHANE report scoring; NASA-TLX; Garmin smartwatches (biometrics) | During simulation |
| Sibley 2018 | Quantitative | Standardised data collection sheet (manual) | During simulation |
| Chang 2022 | Quantitative | Self-assessment of Chemical Disaster Preparedness Inventory (SCDPI); General Self-Efficacy Scale (GSES); demographic form | Pre, 1-week, and 3-weeks post-intervention |
| Foronda 2016 | Mixed | 20-item MCQ test (Qualtrics); debriefing field notes (thematic analysis) | Pre and post |
| Bajow 2016 | Mixed | 25-item MCQ; 9-item Likert questionnaire; open-ended survey items; semi-structured follow-up questionnaire; instructor observation/feedback sheets | Pre, during, and post training |

***Note:*** *For registered protocols (ChiCTR2300072282, NCT06253156, NCT06034184), instruments and timing reflect planned methodology as described in the registration. Outcomes have not yet been reported.*
