# Supplementary material for: Technology-enhanced training for prehospital mass-casualty incident preparedness: A scoping review
Source: PLoS One. 2026 Nov 12;21(11):e0348829. doi: 10.1371/journal.pone.0348829 (PMC13759121; doi:10.1371/journal.pone.0348829)
Supplement: Supplementary file 6 [file pone.0348829.s007.docx]

05 July, 2026

Saima Aleem
Academic Editor
PLOS ONE

Subject: Response to Decision on Manuscript (PONE-D-26-19201)

Title: Technology-enhanced training for prehospital mass-casualty incident preparedness: A scoping review

Dear Dr. Aleem,

I am Dr. Azza Yousif, the corresponding author of the manuscript submitted to PLOS ONE (Manuscript ID: PONE-D-26-19201), entitled “Technology-enhanced training for prehospital mass-casualty incident preparedness: A scoping review.”

Thank you for the time and consideration given to our submission. We would also like to sincerely thank the reviewers for their constructive and insightful feedback, which helped us improve the clarity, methodological transparency, and overall quality of the manuscript.

We have carefully reviewed all comments and revised the manuscript accordingly. We have uploaded the revised manuscript with changes highlighted, a cleaned version, and the required response letter via the submission system.

Our point-by-point responses to the reviewers’ comments are provided below.

Yours sincerely,

Dr. Azza Yousif
On behalf of all authors

**Reviewer Comments to the Author**

**Reviewer #1:**

**This is a scoping review addressing the role of technology in the delivery and assessment of disaster preparedness. This was well written and thoroughly searched. It addresses deficiencies in the existing literature. The choice of scoping review is approriate to explore areas that were undereporter, and inform future practice.
This review abided by the PRISMA-Scr components.
The protocol was registered apriori**

**Response:**

Thank you very much for your positive and encouraging feedback. We appreciate your recognition of the importance of this topic, the appropriateness of the scoping review design, the thoroughness of the search, and our adherence to PRISMA-ScR. We also appreciate your acknowledgment that the protocol was registered a priori.

**Reviewer #2:**

**1. Although quality appraisal is optional in scoping reviews, its absence here is a missed opportunity, especially given that the authors use the findings to propose a new evaluative tier (L2+). Without a formal assessment of the risk of bias or methodological quality of the 28 included studies, the strength of the recommendations—particularly those regarding policy and investment—remains unsubstantiated.**

**Response:**

Thank you for this helpful comment. We agree that conducting a quality appraisal could add value to the study and strengthen the interpretation of the findings. However, in line with the Arksey and O’Malley framework and Levac et al.’s refinements, formal risk-of-bias or methodological quality assessment is not required for scoping reviews, as the aim is to map the extent, nature, and characteristics of the literature rather than determine evidence certainty or pooled effectiveness.

Because of the heterogeneous study designs included in this review, conducting a formal quality appraisal would be challenging. We appreciate this recommendation and will consider it for future systematic reviews or effectiveness-focused studies, but we did not conduct a formal quality appraisal in the present scoping review.

To address your concern transparently and to avoid overstating the findings, we added explicit wording in Section 4.5, first paragraph (p. 37), stating that the review “did not include a formal risk-of-bias appraisal” and that the findings “should be interpreted as a map of the evidence base rather than a judgment of effectiveness certainty.”

**2. The manuscript mentions that screening and data extraction were performed by multiple reviewers. However, it lacks a formal report of inter-rater reliability (e.g., Cohen’s Kappa). Providing this metric is essential for transparency and to confirm the consistency of the study selection and data coding process.**

**Response:**

Thank you very much for this important comment. We appreciate the reviewer’s point regarding transparency and consistency between reviewers during study selection and data charting.

This study was conducted as a scoping review following the methodological framework of Arksey and O’Malley, refined by Levac et al., and reported in accordance with the PRISMA-ScR checklist. We did not calculate Cohen’s kappa because this statistic was not prospectively planned or calculated during the review process. In addition, PRISMA-ScR requires transparent reporting of the process used to select sources of evidence and chart data, but it does not require reporting inter-rater reliability statistics such as Cohen’s kappa.

We agree that formal inter-rater reliability statistics can be useful in some evidence syntheses. However, because Cohen’s kappa was not calculated prospectively, we have not added a retrospective kappa value. Instead, to improve methodological transparency, we clarified the duplicate-review and disagreement-resolution process in Section 2.5, final paragraph (p. 11–12): “The data-charting process was managed through independent duplicate extraction followed by comparison and reconciliation of reviewer datasets. Discrepancies were resolved through discussion, with third-reviewer consultation when needed.”

**3. While the authors propose innovative constructs like “Data Capture Architecture” and “Pedagogical Transparency Gap,” the manuscript lacks a clear coding manual or detailed operational definitions for these terms. It is unclear how these themes were consistently identified across diverse study designs.**

**Response:**

Thank you for this constructive comment. We agree that the analytical constructs require clearer operational definitions and coding rules. To address this, we added **Appendix S2: Operational definitions and rules for study-level construct assignment**, which provides the construct type, operational definition, permitted categories, required evidence, and decision rules for the Technology Function Spectrum, Data Capture Architecture, and Pedagogical Transparency Gap. This appendix clarifies how these constructs were consistently identified and applied across the included studies.

**Reviewer #3: The manuscript deals with one of the most important public health and clinical problem which is mass casualty incident training and assessment globaly which will improve the clinical outcomes of patients and improve the training of disaster medicine nd and the pre hospital preparedeness and management. In general some of the tense use expose your paper to be protocol rather than an outcome of a study.If systematic review is done what specific questions were addressed by this study?**

**Response:**

Thank you for this helpful comment. We revised the manuscript to clarify that this is a scoping review, not a systematic review, and to avoid wording that could make the paper appear to be a protocol rather than a completed review. The aim of this scoping review was to map the extent, nature, and characteristics of the literature on technology-enhanced training for prehospital MCI preparedness, rather than to conduct an effectiveness-focused systematic review.

We also revised the tense throughout the manuscript where needed. The review questions addressed were: (1) what technologies have been used to train and assess healthcare learners and professionals in prehospital MCI preparedness; (2) what learner outcomes and measurement approaches have been reported; and (3) which training design frameworks are reported and how clearly they are described.

**The formating and guideline should be in line with the guideline of the Journal. E.g. The abstract section. The abstract lacks clarity and needs proper revision.**

**Response:**

Thank you for this helpful comment. We revised the abstract to improve clarity, structure, and alignment with the journal format. The revised abstract now presents a clearer Background, Methods, Results, and Conclusions sequence and more explicitly summarizes the review scope, the modified Kirkpatrick mapping, and the principal findings (Abstract, pp. 2–3).

**The introduction is good but didn't defined mass casualty incident and its types, the causes and what were the associated factors, what happens if MCI is not well addressed, moreover, the knowledge gap remains to some extent vacant. For instance some lacks references E.g.line 53 to 56.**

**Response:**

Thank you for this comment. We revised the Introduction to address these points. We added a clear definition of mass-casualty incident (MCI) and described the main causes and categories of MCIs, including natural disasters, human-made disasters, terrorist attacks, transportation incidents, fires, hazardous material exposure, extreme weather events, and large-scale public health emergencies. We also strengthened the explanation of why poorly addressed MCIs can lead to preventable morbidity, mortality, and system failure.

In addition, we revised the knowledge gap paragraph to make the rationale for the review clearer. The revised paragraph now explains that previous reviews focused mainly on extended reality technologies and did not fully map the broader range of digital tools, their educational functions, assessment approaches, training frameworks, evaluation levels, or implementation barriers. We also added supporting references where needed, including the previously under-referenced statements around the challenges of traditional disaster education and MCI training.

**The materials and methods section is again good. However, the inclusion criteria should entail the year 2015 and English language publications. WHY year 2015 is selected?**

**Response:**

Thank you for this helpful comment. We revised Section 2.4.1, first paragraph (pp. 9–10), to state more explicitly that inclusion was limited to studies published from 2015 onward, and we added the rationale for this threshold. The revised paragraph explains that 2015 was selected to capture a contemporary evidence base aligned with the Sendai Framework for Disaster Risk Reduction 2015–2030, the broader uptake of current mobile and immersive technologies, and the consolidation of competency-based and simulation-based medical education. We also made the language restriction explicit in the “Publication characteristics” bullet (“Studies available in English were only eligible”

**Why don’t you include the knowledge, affective and skill domain in the training section.**

**Response:**

Thank you for this comment. The knowledge, affective, and skill domains were already captured within the Kirkpatrick-based outcome classification used in this review. Affective outcomes were captured mainly under Level 1 reaction and related learner-reported outcomes, including satisfaction, engagement, confidence, motivation, attitudes, perceived preparedness, stress, and workload. Knowledge outcomes were captured under Level 2 learning, including knowledge acquisition and protocol understanding. Skill-based outcomes were captured through Level 2+ applied learning and Level 3 behavior-related outcomes, including triage accuracy, triage time, decision-making, communication, protocol adherence, and other performance-based measures.

Therefore, we did not add knowledge, affective, and skill domains as separate training-domain categories, as this would duplicate the existing Kirkpatrick classification rather than add a distinct analytical layer.

**By the way have you used the full framework and its constructs in the research process?**

**Response:**

Thank you for this comment. Yes, the manuscript uses the full Kirkpatrick framework to organize reported outcomes, and this is described in Section 2.5.1 (pp. 12–13) and reflected in Section 3.6 and Table 4 (pp. 25–29). The manuscript also retains the L2+ Applied Learning sub-level to distinguish embedded simulation-based performance assessment from real-world behavioral transfer. This framework guided the extraction, classification, and presentation of training outcomes throughout the review.

**Above all, where is the section that deals with the quality of the papers ?**

**Response:**

Thank you for this comment. We did not include a separate section on the quality of the included papers because this study is a scoping review. In line with the Arksey and O’Malley framework and Levac et al.’s refinements, formal risk-of-bias or methodological quality assessment is not required for scoping reviews, as the aim is to map the extent, nature, and characteristics of the literature rather than determine evidence certainty or pooled effectiveness.

However, to make this limitation explicit, we added clarifying language in Section 4.5, first paragraph (p. 37), stating that the review “did not include a formal risk-of-bias appraisal” and that the findings should be interpreted cautiously as evidence mapping rather than as a statement of effectiveness certainty.

**The results section is again good but the sub gropus lack the sub type of injuries, age and sex distribution of the papers? Don't interprete the results and simply present it in clear, brief and accurate way. Use NB instead of note.**

**Response:**

Thank you for this comment. Subgroups based on injury subtype, age, and sex were not included because this scoping review focused on technology-enhanced training interventions for prehospital MCI preparedness, not on clinical injury cohorts or patient outcomes. Therefore, injury subtype was not applicable as a subgroup variable. Age and sex were also not consistently reported across the included training studies and were not central to the review questions, which focused on technology type, educational function, measurement approach, and training outcomes.

We have revised the Results section to keep the presentation clearer, briefer, and more descriptive, with interpretation reserved for the Discussion. We also replaced “Note” with “NB” where applicable.

**The dicussion covers wide space. Where are the main findings and how were his findings relate each other, where is the critical review and assessment of the studies, focus on the study questions and deliver accordingly. Where are the comparisons? Where are the specific implications? Expand the arguments and explanations of the review findings with up to date references. Try to summerize it shortly and critically and don't repeat the results section in repeated manner. Finally, mention, the strength and the limitations of the study.

Response:**

Thank you for this constructive comment. We substantially revised the Discussion to foreground the main findings, relate them to one another more clearly, compare them with relevant literature, and state the practical implications more explicitly. The section is now organized into focused subsections on the function-measurement nexus (4.1), pedagogical transparency and evaluation depth (4.2), scalability and disaster equity (4.3), future research/reporting needs (4.4), and study limitations/strengths (4.5) (pp. 33–37). The revised opening now states that the review identified “four distinct empirical patterns across the evidence base,” and the Discussion cites additional recent literature to support these comparisons and implications. We also added a dedicated “Limitations and Strengths” subsection and revised the Conclusions accordingly.
