# Supplementary material for: Technology-enhanced training for prehospital mass-casualty incident preparedness: A scoping review
Source: PLoS One. 2026 Nov 12;21(11):e0348829. doi: 10.1371/journal.pone.0348829 (PMC13759121; doi:10.1371/journal.pone.0348829)
Supplement: Supplementary file 7 [file pone.0348829.s008.docx]

22 September, 2026

Saima Aleem
Academic Editor
PLOS ONE

Subject: Response to Decision on Manuscript (PONE-D-26-19201R1)

Title: Technology-enhanced training for prehospital mass-casualty incident preparedness: A scoping review

Dear Dr. Aleem,

I am Dr. Azza Yousif, the corresponding author of the manuscript submitted to PLOS ONE (Manuscript ID: PONE-D-26-19201R1), entitled “Technology-enhanced training for prehospital mass-casualty incident preparedness: A scoping review.”

Thank you for the time and consideration given to our submission. We thank Reviewer 3 for acknowledging our responses to the previous round of review and for the additional recommendations. We have addressed the five comments below. Section references refer to the revised manuscript. Yellow highlighting identifies revised or added text, relocated text, and consequential heading renumbering. References in quoted passages retain the manuscript’s numbering. Our point-by-point responses to the reviewer comments are provided below.

Yours sincerely,

Dr. Azza Yousif
On behalf of all authors

**Reviewer Comments to the Author**

# A. Abstract methods

**Reviewer comment:** A.Abstract: Good but the methods section should be explicitly.

**Response:** We have made the Methods paragraph more explicit by stating the methodological framework and Levac refinements, PRISMA-ScR reporting, English-language eligibility, the 2015–2025 publication window, inclusion of studies and protocols, and the healthcare population and prehospital MCI context. It now states that two reviewers independently screened and extracted data, describes the descriptive and narrative synthesis, and clarifies that Kirkpatrick outcome mapping was undertaken for completed studies. The individual database names remain in the full Methods section. The abstract remains 300 words.

**Location:** Abstract, Methods paragraph.

“Methods: Following Arksey and O'Malley's framework with Levac refinements and PRISMA-ScR reporting, we searched seven databases and supplementary sources for English-language studies and protocols (2015–2025) on technology-enhanced prehospital MCI training for healthcare learners and professionals. Two reviewers independently screened and extracted data. Descriptive and narrative synthesis included modified Kirkpatrick outcome mapping for completed studies, incorporating L2+ (Applied Learning).”

# B. MCI context, rationale, and time period

**Reviewer comment:** B.Introduction: It fails to dectate the when,where and strong rationale of the MCI and the time period.

**Response:** We have added a paragraph defining the review’s focus on preparation for the early prehospital phase of MCI response, before hospital-based care, and specifying the relevant field, ambulance, roadside, and disaster-zone settings. This also makes the international scope and the operational challenge of exceeding local response capacity explicit. A second paragraph states the 2015–2025 publication window and explains the existing rationale for the 2015 starting point: alignment with the Sendai Framework, a focus on contemporary mobile and immersive technologies, and competency-based and simulation-based education. The supporting methodological detail and references remain in Section 2.4.1. It connects this timeframe to the practical need for repeatable training and meaningful competency assessment. The timeframe describes the literature being reviewed, not a restriction on when MCIs occur.

**Location:** Section 1, Introduction, new second and sixth paragraphs.

“The present review concerns preparation for the early prehospital phase of MCI response, before hospital-based care, including field triage, ambulance scenarios, roadside emergencies, and disaster zones. It examines training for healthcare learners and professionals across international settings, where the central challenge is coordinating triage, communication, and resource use when local response capacity is exceeded [1,2].”

“The review covers literature published during 2015–2025. The 2015 starting point aligns the review with the Sendai Framework for Disaster Risk Reduction 2015–2030 and its emphasis on preparedness [10]. The window was selected to focus on contemporary mobile and immersive technologies and competency-based and simulation-based education, as detailed in the eligibility criteria (Section 2.4.1). Within this period, the practical rationale is to identify how technologies support repeatable training and meaningful competency assessment, rather than simply how they reproduce disaster scenarios.”

# C. Methods concision and eligibility criteria

**Reviewer comment:** C.Methods: It is not brief and make the illigiblity criteria intact.

**Response:** We have shortened the Materials and Methods section from 2,152 to 1,676 words, a reduction of approximately 22%, by condensing repetitive explanations of the review design, searching, study selection, data charting, outcome classification, framework extraction, synthesis, analytical constructs, and ethics. These are reporting edits; the review procedures have not been changed.

The five-stage structure, primary and secondary questions, named search sources, PRESS peer review, duplicate screening and extraction, five-study piloting, and the three L2+ classification criteria have been retained. The distinction between descriptive record-based frequencies and outcome mapping for completed studies is also retained. Importantly, Section 2.4.1, including its introductory paragraph and all inclusion and exclusion criteria, is unchanged. This preserves the original population, concept, context, study-design, language, grey-literature, and exclusion rules in one dedicated subsection.

**Location:** Sections 2.1, 2.3, 2.4 (excluding the unchanged Section 2.4.1), 2.5, 2.6, and 2.7.

For example, the shortened study-selection description now reads:

“Two reviewers independently screened titles and abstracts in Covidence against the predefined eligibility criteria. Records considered potentially eligible by both reviewers proceeded to full-text review.”

“Two reviewers independently assessed full texts. Disagreements were resolved by discussion, with a third reviewer making the final decision when consensus was not reached.”

“We recorded full-text exclusion reasons and summarized identification, screening, eligibility assessment, and inclusion in a PRISMA flow diagram.”

# D. Results organization and placement of data types

**Reviewer comment:** D.Results: Good but not systematic and well organized. E.g. The type of data can be put before it is presented immediately after the presentations of major findings.

**Response:** We have moved the former Section 3.9, “Data Collection Methods,” to immediately after Section 3.2, “Study Characteristics,” and retitled it Section 3.3, “Data Types and Collection Methods.” Quantitative, performance-based, qualitative, and mixed-method data sources are therefore described before the technology-specific findings, data-capture analysis, and training outcomes, rather than at the end of the Results.

The Results now proceed from search and screening to study characteristics, data types and collection methods, technologies and their functions, the technology function spectrum, data-capture architecture, training outcomes, pedagogical transparency, and cross-cutting patterns. The affected subsections have been renumbered. The relocated body text, study counts, outcome classifications, tables, and figure captions have not been changed. The entire moved section is highlighted to make the structural revision visible, including text whose wording is unchanged.

**Location:** Section 3.3 and consequential renumbering of Sections 3.4–3.9, including 3.9.1 and 3.9.2.

“3.3. Data Types and Collection Methods”

# E. Clinical and public health practice implications

**Reviewer comment:** E. Well written and should also draw important clinical and public health practice implications?

**Response:** We have added a dedicated subsection, “Clinical and Public Health Practice Implications.” It translates the findings into priorities for task-focused training design, assessment standards, feedback and debriefing, and the distinction between simulation performance and clinical transfer. It also addresses platform access, infrastructure, ongoing costs, local feasibility, and equity in resource-limited settings. The subsection explicitly presents these as training and implementation priorities rather than recommendations based on demonstrated patient benefit, because comparative effectiveness was not established and organizational or patient-level outcomes were not reported. The existing Roadmap and Limitations subsections have only been renumbered to accommodate this addition.

**Location:** New Section 4.4; former Sections 4.4 and 4.5 are now Sections 4.5 and 4.6.

“For clinical practice, the findings suggest that prehospital education programs should specify the tasks to be rehearsed, including triage prioritization, communication, and time-sensitive decision-making, before selecting a training platform. Where feasible, performance records should be linked to an explicit assessment standard and used in feedback and debriefing to identify learning needs. Embedded logs can support this process, but data capture alone should not be interpreted as evidence of better clinical performance. Applied simulation outcomes, including L2+, should be distinguished from evidence of transfer to routine practice or actual MCI response.”

“For public health practice, EMS leaders and preparedness planners should consider access, repeatability, infrastructure, technical support, and ongoing costs alongside instructional and assessment requirements. Screen-based, mobile, or hybrid approaches may warrant local evaluation where immersive systems are difficult to sustain. The absence of studies from low- or lower-middle-income countries means that feasibility, acceptability, and equitable access in these settings cannot be assumed from the present evidence. Training investments should therefore be accompanied by context-specific implementation evaluation [10,11,59,60].”

“These implications are priorities for training design and implementation, not recommendations based on demonstrated patient benefit. The review did not establish comparative effectiveness, and no included study reported organizational or patient-level outcomes. Future evaluations should assess whether improvements in simulated performance are sustained and translate into better clinical and system-level response.”
