# Supplementary material for: Technology-enhanced training for prehospital mass-casualty incident preparedness: A scoping review
Source: PLoS One. 2026 Nov 12;21(11):e0348829. doi: 10.1371/journal.pone.0348829 (PMC13759121; doi:10.1371/journal.pone.0348829)
Supplement: Peer review history

# Peer review: Decision Letter 0

**DOI:** 10.1371/journal.pone.0348829.r001

**Authors:** Saima Aleem

2 Jun 2026

Dear Dr. Yousif,

Thank you for submitting your manuscript to PLOS ONE. After careful consideration, we feel that it has merit but does not fully meet PLOS ONE’s publication criteria as it currently stands. Therefore, we invite you to submit a revised version of the manuscript that addresses the points raised during the review process.

Please submit your revised manuscript by Jul 17 2026 11:59PM. If you will need more time than this to complete your revisions, please reply to this message or contact the journal office at plosone@plos.org. When you're ready to submit your revision, log on to https://www.editorialmanager.com/pone/ and select the 'Submissions Needing Revision' folder to locate your manuscript file.

- A letter that responds to each point raised by the academic editor and reviewer(s). You should upload this letter as a separate file labeled 'Response to Reviewers'.
- A marked-up copy of your manuscript that highlights changes made to the original version. You should upload this as a separate file labeled 'Revised Manuscript with Track Changes'.
- An unmarked version of your revised paper without tracked changes. You should upload this as a separate file labeled 'Manuscript'.

If you would like to make changes to your financial disclosure, please include your updated statement in your cover letter. Guidelines for resubmitting your figure files are available below the reviewer comments at the end of this letter.

If applicable, we recommend that you deposit your laboratory protocols in protocols.io to enhance the reproducibility of your results. Protocols.io assigns your protocol its own identifier (DOI) so that it can be cited independently in the future. For instructions see: https://journals.plos.org/plosone/s/submission-guidelines#loc-laboratory-protocols. Additionally, PLOS ONE offers an option for publishing peer-reviewed Lab Protocol articles, which describe protocols hosted on protocols.io. Read more information on sharing protocols at https://plos.org/protocols?utm_medium=editorial-email&utm_source=authorletters&utm_campaign=protocols.

As the corresponding author, your ORCID iD is verified in the submission system and will appear in the published article. PLOS supports the use of ORCID, and we encourage all coauthors to register for an ORCID iD and use it as well. Please encourage your coauthors to verify their ORCID iD within the submission system before final acceptance, as unverified ORCID iDs will not appear in the published article. *Only*  the individual author can complete the verification step; PLOS staff *cannot*  verify ORCID iDs on behalf of authors.

We look forward to receiving your revised manuscript.

Kind regards,

Saima Aleem

Academic Editor

PLOS One

Journal Requirements:

When submitting your revision, we need you to address these additional requirements.

1. Please ensure that your manuscript meets PLOS ONE's style requirements, including those for file naming. The PLOS ONE style templates can be found at

https://journals.plos.org/plosone/s/file?id=wjVg/PLOSOne_formatting_sample_main_body.pdf and

https://journals.plos.org/plosone/s/file?id=ba62/PLOSOne_formatting_sample_title_authors_affiliations.pdf

2. If the reviewer comments include a recommendation to cite specific previously published works, please review and evaluate these publications to determine whether they are relevant and should be cited. There is no requirement to cite these works unless the editor has indicated otherwise.

[Note: HTML markup is below. Please do not edit.]

Reviewers' comments:

Reviewer's Responses to Questions

**Comments to the Author**

1. Is the manuscript technically sound, and do the data support the conclusions?

Reviewer #1: Yes

Reviewer #2: Partly

Reviewer #3: Partly

**********

2. Has the statistical analysis been performed appropriately and rigorously?

Reviewer #1: Yes

Reviewer #2: Yes

Reviewer #3: Yes

**********

3. Have the authors made all data underlying the findings in their manuscript fully available?

The PLOS Data policy

Reviewer #1: Yes

Reviewer #2: Yes

Reviewer #3: Yes

**********

4. Is the manuscript presented in an intelligible fashion and written in standard English?

Reviewer #1: Yes

Reviewer #2: Yes

Reviewer #3: Yes

**********

Reviewer #1: This is a scoping review addressing the role of technology in the delivery and assessment of disaster preparedness. This was well written and thoroughly searched. It addresses deficiencies in the existing literature. The choice of scoping review is approriate to explore areas that were undre reporter, and inform future practice.

This review abided by the PRISMA-Scr components.

The protocol was registered apriori

Reviewer #2: 1. Although quality appraisal is optional in scoping reviews, its absence here is a missed opportunity, especially given that the authors use the findings to propose a new evaluative tier (L2+). Without a formal assessment of the risk of bias or methodological quality of the 28 included studies, the strength of the recommendations—particularly those regarding policy and investment—remains unsubstantiated.

2. The manuscript mentions that screening and data extraction were performed by multiple reviewers. However, it lacks a formal report of inter-rater reliability (e.g., Cohen’s Kappa). Providing this metric is essential for transparency and to confirm the consistency of the study selection and data coding process.

3. While the authors propose innovative constructs like “Data Capture Architecture” and “Pedagogical Transparency Gap,” the manuscript lacks a clear coding manual or detailed operational definitions for these terms. It is unclear how these themes were consistently identified across diverse study designs.

Reviewer #3: The manuscript deals with one of the most important public health and clinical problem which is mass casualty incident training and assessment globaly which will improve the clinical outcomes of patients and improve the training of disaster medicine nd and the pre hospital preparedeness and management. In general some of the tense use expose your paper to be protocol rather than an outcome of a study.If systematic review is done what specific questions were addressed by this study?

The formating and guideline should be in line with the guideline of the Journal. E.g. The abstract section. The abstract lacks clarity and needs proper revision. The introduction is good but didn't defined mass casualty incident and its types, the causes and what were the associated factors, what happens if MCI is not well addressed, moreover, the knowledge gap remains to some extent vacant. For instance some lacks references E.g.line 53 to 56. The materials and methods section is again good. However, the inclusion criteria should entail the year 2015 and English language publications. WHY year 2015 is selected? Why don’t you include the knowledge, affective and skill domain in the training section. By the way have you used the full framework and its constructs in the research process? Above all, where is the section that deals with the quality of the papers ? The results section is again good but the sub gropus lack the sub type of injuries, age and sex distribution of the papers? Don't interprete the results and simply present it in clear, brief and accurate way. Use NB instead of note. The dicussion covers wide space. Where are the main findings and how were his findings relate each other, where is the critical review and assessment of the studies, focus on the study questions and deliver accordingly. Where are the comparisons? Where are the specific implications? Expand the arguments and explanations of the review findings with up to date references. Try to summerize it shortly and critically and don't repeat the results section in repeated manner. Finally, mention, the strength and the limitations of the study.

Regards,

**********

what does this mean?). If published, this will include your full peer review and any attached files.

If you choose “no”, your identity will remain anonymous but your review may still be made public.

**Do you want your identity to be public for this peer review?**  For information about this choice, including consent withdrawal, please see our Privacy Policy

Reviewer #1: **Yes:**  Noora Alshahwani

Reviewer #2: **Yes:**  Ehsan Abedini

Reviewer #3: No

**********

[NOTE: If reviewer comments were submitted as an attachment file, they will be attached to this email and accessible via the submission site. Please log into your account, locate the manuscript record, and check for the action link "View Attachments". If this link does not appear, there are no attachment files.]

To ensure your figures meet our technical requirements, please review our figure guidelines: https://journals.plos.org/plosone/s/figures

You may also use PLOS’s free figure tool, NAAS, to help you prepare publication quality figures: https://journals.plos.org/plosone/s/figures#loc-tools-for-figure-preparation.

NAAS will assess whether your figures meet our technical requirements by comparing each figure against our figure specifications.

# Author comment: Author response to Decision Letter 1

**DOI:** 10.1371/journal.pone.0348829.r002

4 Jul 2026

Reviewer Comments to the Author

Reviewer #1:

This is a scoping review addressing the role of technology in the delivery and assessment of disaster preparedness. This was well written and thoroughly searched. It addresses deficiencies in the existing literature. The choice of scoping review is approriate to explore areas that were undereporter, and inform future practice.

This review abided by the PRISMA-Scr components.

The protocol was registered apriori

Response:

Thank you very much for your positive and encouraging feedback. We appreciate your recognition of the importance of this topic, the appropriateness of the scoping review design, the thoroughness of the search, and our adherence to PRISMA-ScR. We also appreciate your acknowledgment that the protocol was registered a priori.

Reviewer #2:

1. Although quality appraisal is optional in scoping reviews, its absence here is a missed opportunity, especially given that the authors use the findings to propose a new evaluative tier (L2+). Without a formal assessment of the risk of bias or methodological quality of the 28 included studies, the strength of the recommendations—particularly those regarding policy and investment—remains unsubstantiated.

Response:

Thank you for this helpful comment. We agree that conducting a quality appraisal could add value to the study and strengthen the interpretation of the findings. However, in line with the Arksey and O’Malley framework and Levac et al.’s refinements, formal risk-of-bias or methodological quality assessment is not required for scoping reviews, as the aim is to map the extent, nature, and characteristics of the literature rather than determine evidence certainty or pooled effectiveness.

Because of the heterogeneous study designs included in this review, conducting a formal quality appraisal would be challenging. We appreciate this recommendation and will consider it for future systematic reviews or effectiveness-focused studies, but we did not conduct a formal quality appraisal in the present scoping review.

To address your concern transparently and to avoid overstating the findings, we added explicit wording in Section 4.5, first paragraph (p. 37), stating that the review “did not include a formal risk-of-bias appraisal” and that the findings “should be interpreted as a map of the evidence base rather than a judgment of effectiveness certainty.”

2. The manuscript mentions that screening and data extraction were performed by multiple reviewers. However, it lacks a formal report of inter-rater reliability (e.g., Cohen’s Kappa). Providing this metric is essential for transparency and to confirm the consistency of the study selection and data coding process.

Response:

Thank you very much for this important comment. We appreciate the reviewer’s point regarding transparency and consistency between reviewers during study selection and data charting.

This study was conducted as a scoping review following the methodological framework of Arksey and O’Malley, refined by Levac et al., and reported in accordance with the PRISMA-ScR checklist. We did not calculate Cohen’s kappa because this statistic was not prospectively planned or calculated during the review process. In addition, PRISMA-ScR requires transparent reporting of the process used to select sources of evidence and chart data, but it does not require reporting inter-rater reliability statistics such as Cohen’s kappa.

We agree that formal inter-rater reliability statistics can be useful in some evidence syntheses. However, because Cohen’s kappa was not calculated prospectively, we have not added a retrospective kappa value. Instead, to improve methodological transparency, we clarified the duplicate-review and disagreement-resolution process in Section 2.5, final paragraph (p. 11–12): “The data-charting process was managed through independent duplicate extraction followed by comparison and reconciliation of reviewer datasets. Discrepancies were resolved through discussion, with third-reviewer consultation when needed.”

3. While the authors propose innovative constructs like “Data Capture Architecture” and “Pedagogical Transparency Gap,” the manuscript lacks a clear coding manual or detailed operational definitions for these terms. It is unclear how these themes were consistently identified across diverse study designs.

Response:

Thank you for this constructive comment. We agree that the analytical constructs require clearer operational definitions and coding rules. To address this, we added Appendix S2: Operational definitions and rules for study-level construct assignment, which provides the construct type, operational definition, permitted categories, required evidence, and decision rules for the Technology Function Spectrum, Data Capture Architecture, and Pedagogical Transparency Gap. This appendix clarifies how these constructs were consistently identified and applied across the included studies.

Reviewer #3:

The manuscript deals with one of the most important public health and clinical problem which is mass casualty incident training and assessment globaly which will improve the clinical outcomes of patients and improve the training of disaster medicine nd and the pre hospital preparedeness and management. In general some of the tense use expose your paper to be protocol rather than an outcome of a study.If systematic review is done what specific questions were addressed by this study?

Response:

Thank you for this helpful comment. We revised the manuscript to clarify that this is a scoping review, not a systematic review, and to avoid wording that could make the paper appear to be a protocol rather than a completed review. The aim of this scoping review was to map the extent, nature, and characteristics of the literature on technology-enhanced training for prehospital MCI preparedness, rather than to conduct an effectiveness-focused systematic review.

We also revised the tense throughout the manuscript where needed. The review questions addressed were: (1) what technologies have been used to train and assess healthcare learners and professionals in prehospital MCI preparedness; (2) what learner outcomes and measurement approaches have been reported; and (3) which training design frameworks are reported and how clearly they are described.

The formating and guideline should be in line with the guideline of the Journal. E.g. The abstract section. The abstract lacks clarity and needs proper revision.

Response:

Thank you for this helpful comment. We revised the abstract to improve clarity, structure, and alignment with the journal format. The revised abstract now presents a clearer Background, Methods, Results, and Conclusions sequence and more explicitly summarizes the review scope, the modified Kirkpatrick mapping, and the principal findings (Abstract, pp. 2–3).

The introduction is good but didn't defined mass casualty incident and its types, the causes and what were the associated factors, what happens if MCI is not well addressed, moreover, the knowledge gap remains to some extent vacant. For instance some lacks references E.g.line 53 to 56.

Response:

Thank you for this comment. We revised the Introduction to address these points. We added a clear definition of mass-casualty incident (MCI) and described the main causes and categories of MCIs, including natural disasters, human-made disasters, terrorist attacks, transportation incidents, fires, hazardous material exposure, extreme weather events, and large-scale public health emergencies. We also strengthened the explanation of why poorly addressed MCIs can lead to preventable morbidity, mortality, and system failure.

In addition, we revised the knowledge gap paragraph to make the rationale for the review clearer. The revised paragraph now explains that previous reviews focused mainly on extended reality technologies and did not fully map the broader range of digital tools, their educational functions, assessment approaches, training frameworks, evaluation levels, or implementation barriers. We also added supporting references where needed, including the previously under-referenced statements around the challenges of traditional disaster education and MCI training.

The materials and methods section is again good. However, the inclusion criteria should entail the year 2015 and English language publications. WHY year 2015 is selected?

Response:

Thank you for this helpful comment. We revised Section 2.4.1, first paragraph (pp. 9–10), to state more explicitly that inclusion was limited to studies published from 2015 onward, and we added the rationale for this threshold. The revised paragraph explains that 2015 was selected to capture a contemporary evidence base aligned with the Sendai Framework for Disaster Risk Reduction 2015–2030, the broader uptake of current mobile and immersive technologies, and the consolidation of competency-based and simulation-based medical education. We also made the language restriction explicit in the “Publication characteristics” bullet (“Studies available in English were only eligible”

Why don’t you include the knowledge, affective and skill domain in the training section.

Response:

Thank you for this comment. The knowledge, affective, and skill domains were already captured within the Kirkpatrick-based outcome classification used in this review. Affective outcomes were captured mainly under Level 1 reaction and related learner-reported outcomes, including satisfaction, engagement, confidence, motivation, attitudes, perceived preparedness, stress, and workload. Knowledge outcomes were captured under Level 2 learning, including knowledge acquisition and protocol understanding. Skill-based outcomes were captured through Level 2+ applied learning and Level 3 behavior-related outcomes, including triage accuracy, triage time, decision-making, communication, protocol adherence, and other performance-based measures.

Therefore, we did not add knowledge, affective, and skill domains as separate training-domain categories, as this would duplicate the existing Kirkpatrick classification rather than add a distinct analytical layer.

By the way have you used the full framework and its constructs in the research process?

Response:

Thank you for this comment. Yes, the manuscript uses the full Kirkpatrick framework to organize reported outcomes, and this is described in Section 2.5.1 (pp. 12–13) and reflected in Section 3.6 and Table 4 (pp. 25–29). The manuscript also retains the L2+ Applied Learning sub-level to distinguish embedded simulation-based performance assessment from real-world behavioral transfer. This framework guided the extraction, classification, and presentation of training outcomes throughout the review.

Above all, where is the section that deals with the quality of the papers ?

Response:

Thank you for this comment. We did not include a separate section on the quality of the included papers because this study is a scoping review. In line with the Arksey and O’Malley framework and Levac et al.’s refinements, formal risk-of-bias or methodological quality assessment is not required for scoping reviews, as the aim is to map the extent, nature, and characteristics of the literature rather than determine evidence certainty or pooled effectiveness.

However, to make this limitation explicit, we added clarifying language in Section 4.5, first paragraph (p. 37), stating that the review “did not include a formal risk-of-bias appraisal” and that the findings should be interpreted cautiously as evidence mapping rather than as a statement of effectiveness certainty.

The results section is again good but the sub gropus lack the sub type of injuries, age and sex distribution of the papers? Don't interprete the results and simply present it in clear, brief and accurate way. Use NB instead of note.

Response:

Thank you for this comment. Subgroups based on injury subtype, age, and sex were not included because this scoping review focused on technology-enhanced training interventions for prehospital MCI preparedness, not on clinical injury cohorts or patient outcomes. Therefore, injury subtype was not applicable as a subgroup variable. Age and sex were also not consistently reported across the included training studies and were not central to the review questions, which focused on technology type, educational function, measurement approach, and training outcomes.

We have revised the Results section to keep the presentation clearer, briefer, and more descriptive, with interpretation reserved for the Discussion. We also replaced “Note” with “NB” where applicable.

The dicussion covers wide space. Where are the main findings and how were his findings relate each other, where is the critical review and assessment of the studies, focus on the study questions and deliver accordingly. Where are the comparisons? Where are the specific implications? Expand the arguments and explanations of the review findings with up to date references. Try to summerize it shortly and critically and don't repeat the results section in repeated manner. Finally, mention, the strength and the limitations of the study.

Response:

Thank you for this constructive comment. We substantially revised the Discussion to foreground the main findings, relate them to one another more clearly, compare them with relevant literature, and state the practical implications more explicitly. The section is now organized into focused subsections on the function-measurement nexus (4.1), pedagogical transparency and evaluation depth (4.2), scalability and disaster equity (4.3), future research/reporting needs (4.4), and study limitations/strengths (4.5) (pp. 33–37). The revised opening now states that the review identified “four distinct empirical patterns across the evidence base,” and the Discussion cites additional recent literature to support these comparisons and implications. We also added a dedicated “Limitations and Strengths” subsection and revised the Conclusions accordingly.

Attachment Submitted filename: Response letter.docx

# Peer review: Decision Letter 1

**DOI:** 10.1371/journal.pone.0348829.r003

**Authors:** Saima Aleem

16 Sep 2026

Dear Dr. Yousif,

Thank you for submitting your manuscript to PLOS One. After careful consideration, we feel that it has merit but does not fully meet PLOS One’s publication criteria as it currently stands. Therefore, we invite you to submit a revised version of the manuscript that addresses the points raised during the review process.

Please submit your revised manuscript by Oct 31 2026 11:59PM. If you will need more time than this to complete your revisions, please reply to this message or contact the journal office at plosone@plos.org. When you're ready to submit your revision, log on to https://www.editorialmanager.com/pone/ and select the 'Submissions Needing Revision' folder to locate your manuscript file.

- A letter that responds to each point raised by the academic editor and reviewer(s). You should upload this letter as a separate file labeled 'Response to Reviewers'.
- A marked-up copy of your manuscript that highlights changes made to the original version. You should upload this as a separate file labeled 'Revised Manuscript with Track Changes'.
- An unmarked version of your revised paper without tracked changes. You should upload this as a separate file labeled 'Manuscript'.

If you would like to make changes to your financial disclosure, please include your updated statement in your cover letter. Guidelines for resubmitting your figure files are available below the reviewer comments at the end of this letter.

If applicable, we recommend that you deposit your laboratory protocols in protocols.io to enhance the reproducibility of your results. Protocols.io assigns your protocol its own identifier (DOI) so that it can be cited independently in the future. For instructions see: https://journals.plos.org/plosone/s/submission-guidelines#loc-laboratory-protocols. Additionally, PLOS One offers an option for publishing peer-reviewed Lab Protocol articles, which describe protocols hosted on protocols.io. Read more information on sharing protocols at https://plos.org/protocols?utm_medium=editorial-email&utm_source=authorletters&utm_campaign=protocols.

As the corresponding author, your ORCID iD is verified in the submission system and will appear in the published article. PLOS supports the use of ORCID, and we encourage all coauthors to register for an ORCID iD and use it as well. Please encourage your coauthors to verify their ORCID iD within the submission system before final acceptance, as unverified ORCID iDs will not appear in the published article. *Only*  the individual author can complete the verification step; PLOS staff *cannot*  verify ORCID iDs on behalf of authors.

We look forward to receiving your revised manuscript.

Kind regards,

Saima Aleem

Academic Editor

PLOS One

Journal Requirements:

If the reviewer comments include a recommendation to cite specific previously published works, please review and evaluate these publications to determine whether they are relevant and should be cited. There is no requirement to cite these works unless the editor has indicated otherwise.

Please review your reference list to ensure that it is complete and correct. If you have cited papers that have been retracted, please include the rationale for doing so in the manuscript text, or remove these references and replace them with relevant current references. Any changes to the reference list should be mentioned in the rebuttal letter that accompanies your revised manuscript. If you need to cite a retracted article, indicate the article’s retracted status in the References list and also include a citation and full reference for the retraction notice.

[Note: HTML markup is below. Please do not edit.]

Reviewers' comments:

Reviewer's Responses to Questions

**Comments to the Author**

Reviewer #3: All comments have been addressed

**********

2. Is the manuscript technically sound, and do the data support the conclusions?

Reviewer #3: Partly

**********

3. Has the statistical analysis been performed appropriately and rigorously?

Reviewer #3: N/A

**********

4. Have the authors made all data underlying the findings in their manuscript fully available?

The PLOS Data policy

Reviewer #3: Yes

**********

5. Is the manuscript presented in an intelligible fashion and written in standard English?

Reviewer #3: Yes

**********

Reviewer #3: We acknowledge the authors for addressing the comments given in the previous version.

A.Abstract: Good but the methods section should be explicitly.

B.Introduction: It fails to dectate the when,where and strong rationale of the MCI and the time period.

C.Methods: It is not brief and make the illigiblity criteria intact.

D.Results: Good but not systematic and well organized. E.g. The type of data can be put before it is presented immediately after the presentations of major findings.

E. Well written and should also draw important clinical and public health practice implications?

Regards,

**********

what does this mean?). If published, this will include your full peer review and any attached files.

If you choose “no”, your identity will remain anonymous but your review may still be made public.

**Do you want your identity to be public for this peer review?**  For information about this choice, including consent withdrawal, please see our Privacy Policy

Reviewer #3: No

**********

[NOTE: If reviewer comments were submitted as an attachment file, they will be attached to this email and accessible via the submission site. Please log into your account, locate the manuscript record, and check for the action link "View Attachments". If this link does not appear, there are no attachment files.]

To ensure your figures meet our technical requirements, please review our figure guidelines: https://journals.plos.org/plosone/s/figures

You may also use PLOS’s free figure tool, NAAS, to help you prepare publication quality figures: https://journals.plos.org/plosone/s/figures#loc-tools-for-figure-preparation.

NAAS will assess whether your figures meet our technical requirements by comparing each figure against our figure specifications.

# Author comment: Author response to Decision Letter 2

**DOI:** 10.1371/journal.pone.0348829.r004

21 Sep 2026

A. Abstract methods

Reviewer comment: A.Abstract: Good but the methods section should be explicitly.

Response: We have made the Methods paragraph more explicit by stating the methodological framework and Levac refinements, PRISMA-ScR reporting, English-language eligibility, the 2015–2025 publication window, inclusion of studies and protocols, and the healthcare population and prehospital MCI context. It now states that two reviewers independently screened and extracted data, describes the descriptive and narrative synthesis, and clarifies that Kirkpatrick outcome mapping was undertaken for completed studies. The individual database names remain in the full Methods section. The abstract remains 300 words.

Location: Abstract, Methods paragraph.

“Methods: Following Arksey and O'Malley's framework with Levac refinements and PRISMA-ScR reporting, we searched seven databases and supplementary sources for English-language studies and protocols (2015–2025) on technology-enhanced prehospital MCI training for healthcare learners and professionals. Two reviewers independently screened and extracted data. Descriptive and narrative synthesis included modified Kirkpatrick outcome mapping for completed studies, incorporating L2+ (Applied Learning).”

B. MCI context, rationale, and time period

Reviewer comment: B.Introduction: It fails to dectate the when,where and strong rationale of the MCI and the time period.

Response: We have added a paragraph defining the review’s focus on preparation for the early prehospital phase of MCI response, before hospital-based care, and specifying the relevant field, ambulance, roadside, and disaster-zone settings. This also makes the international scope and the operational challenge of exceeding local response capacity explicit. A second paragraph states the 2015–2025 publication window and explains the existing rationale for the 2015 starting point: alignment with the Sendai Framework, a focus on contemporary mobile and immersive technologies, and competency-based and simulation-based education. The supporting methodological detail and references remain in Section 2.4.1. It connects this timeframe to the practical need for repeatable training and meaningful competency assessment. The timeframe describes the literature being reviewed, not a restriction on when MCIs occur.

Location: Section 1, Introduction, new second and sixth paragraphs.

“The present review concerns preparation for the early prehospital phase of MCI response, before hospital-based care, including field triage, ambulance scenarios, roadside emergencies, and disaster zones. It examines training for healthcare learners and professionals across international settings, where the central challenge is coordinating triage, communication, and resource use when local response capacity is exceeded [1,2].”

“The review covers literature published during 2015–2025. The 2015 starting point aligns the review with the Sendai Framework for Disaster Risk Reduction 2015–2030 and its emphasis on preparedness [10]. The window was selected to focus on contemporary mobile and immersive technologies and competency-based and simulation-based education, as detailed in the eligibility criteria (Section 2.4.1). Within this period, the practical rationale is to identify how technologies support repeatable training and meaningful competency assessment, rather than simply how they reproduce disaster scenarios.”

C. Methods concision and eligibility criteria

Reviewer comment: C.Methods: It is not brief and make the illigiblity criteria intact.

Response: We have shortened the Materials and Methods section from 2,152 to 1,676 words, a reduction of approximately 22%, by condensing repetitive explanations of the review design, searching, study selection, data charting, outcome classification, framework extraction, synthesis, analytical constructs, and ethics. These are reporting edits; the review procedures have not been changed.

The five-stage structure, primary and secondary questions, named search sources, PRESS peer review, duplicate screening and extraction, five-study piloting, and the three L2+ classification criteria have been retained. The distinction between descriptive record-based frequencies and outcome mapping for completed studies is also retained. Importantly, Section 2.4.1, including its introductory paragraph and all inclusion and exclusion criteria, is unchanged. This preserves the original population, concept, context, study-design, language, grey-literature, and exclusion rules in one dedicated subsection.

Location: Sections 2.1, 2.3, 2.4 (excluding the unchanged Section 2.4.1), 2.5, 2.6, and 2.7.

For example, the shortened study-selection description now reads:

“Two reviewers independently screened titles and abstracts in Covidence against the predefined eligibility criteria. Records considered potentially eligible by both reviewers proceeded to full-text review.”

“Two reviewers independently assessed full texts. Disagreements were resolved by discussion, with a third reviewer making the final decision when consensus was not reached.”

“We recorded full-text exclusion reasons and summarized identification, screening, eligibility assessment, and inclusion in a PRISMA flow diagram.”

D. Results organization and placement of data types

Reviewer comment: D.Results: Good but not systematic and well organized. E.g. The type of data can be put before it is presented immediately after the presentations of major findings.

Response: We have moved the former Section 3.9, “Data Collection Methods,” to immediately after Section 3.2, “Study Characteristics,” and retitled it Section 3.3, “Data Types and Collection Methods.” Quantitative, performance-based, qualitative, and mixed-method data sources are therefore described before the technology-specific findings, data-capture analysis, and training outcomes, rather than at the end of the Results.

The Results now proceed from search and screening to study characteristics, data types and collection methods, technologies and their functions, the technology function spectrum, data-capture architecture, training outcomes, pedagogical transparency, and cross-cutting patterns. The affected subsections have been renumbered. The relocated body text, study counts, outcome classifications, tables, and figure captions have not been changed. The entire moved section is highlighted to make the structural revision visible, including text whose wording is unchanged.

Location: Section 3.3 and consequential renumbering of Sections 3.4–3.9, including 3.9.1 and 3.9.2.

“3.3. Data Types and Collection Methods”

E. Clinical and public health practice implications

Reviewer comment: E. Well written and should also draw important clinical and public health practice implications?

Response: We have added a dedicated subsection, “Clinical and Public Health Practice Implications.” It translates the findings into priorities for task-focused training design, assessment standards, feedback and debriefing, and the distinction between simulation performance and clinical transfer. It also addresses platform access, infrastructure, ongoing costs, local feasibility, and equity in resource-limited settings. The subsection explicitly presents these as training and implementation priorities rather than recommendations based on demonstrated patient benefit, because comparative effectiveness was not established and organizational or patient-level outcomes were not reported. The existing Roadmap and Limitations subsections have only been renumbered to accommodate this addition.

Location: New Section 4.4; former Sections 4.4 and 4.5 are now Sections 4.5 and 4.6.

“For clinical practice, the findings suggest that prehospital education programs should specify the tasks to be rehearsed, including triage prioritization, communication, and time-sensitive decision-making, before selecting a training platform. Where feasible, performance records should be linked to an explicit assessment standard and used in feedback and debriefing to identify learning needs. Embedded logs can support this process, but data capture alone should not be interpreted as evidence of better clinical performance. Applied simulation outcomes, including L2+, should be distinguished from evidence of transfer to routine practice or actual MCI response.”

“For public health practice, EMS leaders and preparedness planners should consider access, repeatability, infrastructure, technical support, and ongoing costs alongside instructional and assessment requirements. Screen-based, mobile, or hybrid approaches may warrant local evaluation where immersive systems are difficult to sustain. The absence of studies from low- or lower-middle-income countries means that feasibility, acceptability, and equitable access in these settings cannot be assumed from the present evidence. Training investments should therefore be accompanied by context-specific implementation evaluation [10,11,59,60].”

“These implications are priorities for training design and implementation, not recommendations based on demonstrated patient benefit. The review did not establish comparative effectiveness, and no included study reported organizational or patient-level outcomes. Future evaluations should assess whether improvements in simulated performance are sustained and translate into better clinical and system-level response.”

Attachment Submitted filename: Response to Reviewers.docx

# Peer review: Decision Letter 2

**DOI:** 10.1371/journal.pone.0348829.r005

**Authors:** Saima Aleem

21 Oct 2026

Technology-enhanced training for prehospital mass-casualty incident preparedness: A scoping review

PONE-D-26-19201R2

Dear Dr. Yousif,

We’re pleased to inform you that your manuscript has been judged scientifically suitable for publication and will be formally accepted for publication once it meets all outstanding technical requirements.

Following a careful assessment of the revised manuscript and reviewers’ comments, I am satisfied that the authors have adequately addressed the substantive concerns raised during two peer review rounds.

The revisions have significantly improved the manuscript’s methodological clarity, transparency, presentation of findings, and discussion of implications. While the remaining reviewer suggestions mentioned at the end of this email are appreciated and constructive, I consider the manuscript sufficiently revised and that an additional round of review is not necessary.

Within one week, you’ll receive an e-mail detailing the required amendments. When these have been addressed, you’ll receive a formal acceptance letter and your manuscript will be scheduled for publication.

An invoice will be generated when your article is formally accepted. Please note, if your institution has a publishing partnership with PLOS and your article meets the relevant criteria, all or part of your publication costs will be covered. Please make sure your user information is up-to-date by logging into Editorial Manager at Editorial Manager® and clicking the ‘Update My Information' link at the top of the page. For questions related to billing, please contact billing support.

If your institution or institutions have a press office, please notify them about your upcoming paper to help maximize its impact. If they’ll be preparing press materials, please inform our press team as soon as possible -- no later than 48 hours after receiving the formal acceptance. Your manuscript will remain under strict press embargo until 2 pm Eastern Time on the date of publication. For more information, please contact onepress@plos.org.

Kind regards,

Saima Aleem

Academic Editor

PLOS One

Additional Editor Comments (optional):

Reviewers' comments:

Reviewer's Responses to Questions

**Comments to the Author**

Reviewer #3: All comments have been addressed

**********

2. Is the manuscript technically sound, and do the data support the conclusions?

Reviewer #3: Partly

**********

3. Has the statistical analysis been performed appropriately and rigorously?

Reviewer #3: Yes

**********

4. Have the authors made all data underlying the findings in their manuscript fully available?

The PLOS Data policy

Reviewer #3: Yes

**********

5. Is the manuscript presented in an intelligible fashion and written in standard English?

Reviewer #3: Yes

**********

Reviewer #3: We acknowledge the authors for revising based on the given comments. The abstract is relatively good but lacks clarity again.The introduction again needs brief revision. The methods section fails to explain in detail the methods amd its approaches. How was th quality maintained? Check k the sources and maintain consistency with the objective of the study. The results should align itself with the objective and the methods section. The discussion is weak amd try to strengthen it by drawing focused amd important implications. Try to explain well the conclusion specifically. The recommendation should be specific and focused.

Regards,

**********

what does this mean?). If published, this will include your full peer review and any attached files.

If you choose “no”, your identity will remain anonymous but your review may still be made public.

**Do you want your identity to be public for this peer review?**  For information about this choice, including consent withdrawal, please see our Privacy Policy

Reviewer #3: No

**********

# Editor report: Acceptance letter

**DOI:** 10.1371/journal.pone.0348829.r006

**Authors:** Saima Aleem

PONE-D-26-19201R2

PLOS One

Dear Dr. Yousif,

I'm pleased to inform you that your manuscript has been deemed suitable for publication in PLOS One. Congratulations! Your manuscript is now being handed over to our production team.

At this stage, our production department will prepare your paper for publication. This includes ensuring the following:

* All references, tables, and figures are properly cited

* All relevant supporting information is included in the manuscript submission,

* There are no issues that prevent the paper from being properly typeset

You will receive further instructions from the production team, including instructions on how to review your proof when it is ready. Please keep in mind that we are working through a large volume of accepted articles, so please give us a few days to review your paper and let you know the next and final steps.

Lastly, if your institution or institutions have a press office, please let them know about your upcoming paper now to help maximize its impact. If they'll be preparing press materials, please inform our press team within the next 48 hours. Your manuscript will remain under strict press embargo until 2 pm Eastern Time on the date of publication. For more information, please contact onepress@plos.org.

You will receive an invoice from PLOS for your publication fee after your manuscript has reached the completed accept phase. If you receive an email requesting payment before acceptance or for any other service, this may be a phishing scheme. Learn how to identify phishing emails and protect your accounts at https://explore.plos.org/phishing.

If we can help with anything else, please email us at customercare@plos.org.

Thank you for submitting your work to PLOS One and supporting open access.

Kind regards,

PLOS One Editorial Office Staff

on behalf of

Dr. Saima Aleem

Academic Editor

PLOS One
